# Metabolomic Analysis of *Heliopsis longipes* Roots Across Phenological Stages

**DOI:** 10.3390/molecules31122114

**Published:** 2026-06-16

**Authors:** Victoria Ruiz-Castillo, Ramón Gerardo Guevara-González, César Ibarra-Alvarado, Pedro Alberto Vázquez-Landaverde, Eduardo Rodríguez de San Miguel, Pablo Aguilar-Rodríguez, Martha Elena García-Aguilera, Nuria Esturau-Escofet, Alejandra Rojas-Molina

**Affiliations:** 1 Posgrado en Ciencias Químico Biológicas, Facultad de Química, Universidad Autónoma de Querétaro, Cerro de las Campanas S/N, Santiago de Querétaro C.P. 76010, Querétaro, Mexico; 2 Grupo de Ingeniería de Biosistemas, Facultad de Ingeniería, Universidad Autónoma de Querétaro, Campus Amazcala. Carr. Chichimequillas-Amazcala Km 1 2/N, Amazcala, El Márques C.P. 76265, Querétaro, Mexico; ramon.guevara@uaq.mx; 3 Laboratorio de Investigación Química y Farmacológica de Productos Naturales, Facultad de Química, Universidad Autónoma de Querétaro, Cerro de las Campanas S/N, Santiago de Querétaro C.P. 76010, Querétaro, Mexico; cibarra@uaq.mx; 4 Centro de Investigación en Ciencia Aplicada y Tecnológica Avanzada del Instituto Politécnico Nacional, Unidad Querétaro, Cerro Blanco 141, Colinas del Cimatario, Santiago de Querétaro C.P. 76090, Querétaro, Mexico; pavazquez@ipn.mx; 5 Departamento de Química Analítica, Facultad de Química, Universidad Nacional Autónoma de México, Circuito Escolar S/N, Ciudad Universitaria, Coyoacán, Ciudad de México C.P. 04510, Mexico; erdsmg@unam.mx; 6 Laboratorio Universitario de Resonancia Magnética Nuclear, Instituto de Química, Universidad Nacional Autónoma de México, Circuito Exterior S/N, Ciudad Universitaria, Coyoacán, Ciudad de México C.P. 04510, Mexico

**Keywords:** *Heliopsis longipes*, alkamides, metabolomics, ^1^H NMR spectroscopy, GC-MS, vasodilatory activity

## Abstract

*Heliopsis longipes*, commonly known as chilcuague, is a Mexican medicinal plant recognized for its diverse biological activities, largely attributed to its alkamide content, particularly affinin. However, metabolic variations associated with plant development remain poorly understood. This study evaluated the influence of phenological stage on the phytochemical profile and vasodilatory activity of *H. longipes* roots. Dichloromethane root extracts from plants at different developmental stages were analyzed using metabolomics based on ^1^H NMR spectroscopy, complemented by GC–MS profiling. Major alkamides were isolated and structurally characterized as analytical standards. Notably, three alkamides, *N*-isobutylundeca-2(*E*)-en-8,10-diynamide, *N*-isobutylundeca-3(*E*)-en-8,10-diynamide, and *N*-isobutyl-2(*E*),4(*Z*)-undecadiene-8,10-diynamide, are reported for the first time in *H. longipes* roots. Multivariate analyses (PCA and OPLS-DA) revealed significant stage-dependent metabolic variation, particularly in affinin. The lack of correlation between valine decarboxylase activity and affinin levels suggests additional regulatory steps in its biosynthesis. Vasodilatory activity increased during development, reaching maximum effect during fructification and defoliation stages, with no significant differences between them. These findings highlight the impact of phenological stage on alkamide production and bioactivity, providing a basis for optimizing cultivation and harvest timing.

## 1. Introduction

*Heliopsis longipes*, commonly known as chilcuague, is widely used in Mexico for culinary, insecticidal, and medicinal purposes [1]. It has been demonstrated that organic extracts prepared from the roots of this plant induce antimicrobial [2], antifungal, bacteriostatic [3], anti-inflammatory [4]; and analgesic effects [5,6]. Recently, our research group reported that the dichloromethane and ethanolic extracts of *H. longipes* roots exert vasodilatory [7] and antihypertensive activities [8], which have been attributed to the presence of affinin or spilanthol. Affinin-induced vasodilation was found to involve multiple signaling pathways, including NO/cGMP, CO/cGMP, H_2_S/K_ATP_, and PGI_2_/cAMP [7]. The compound also engaged CB1 receptors and TRPA1 and TRPV1 channels and inhibited L-type calcium channels [9].

Affinin is a compound that has great economic importance in the food, cosmetic, and pharmaceutical industry [10] and to date, 91 products containing affinin have been patented, including hygiene articles, such as toothpaste, oral analgesic (Buccaldol), antibacterial gels (Indolphar^®^), and anti-aging products (Gatuline^®^, SYN^®^-COLL, ChroNOline^TM^) [11].

Regarding their chemical composition, plants of the *Heliopsis* genus are characterized by synthesizing guaianolide-type sesquiterpene lactones and homoditerpenes in their aerial parts [12], while aliphatic and acetylenic alkamides [1] and lignans are biosynthesized in the root [13]. Chemical studies carried out on *H. longipes* have focused on the root, whose main constituents are aliphatic alkamides such as affinin, longipinamide A, longipenamide A, longipenamide B, *N*-(2-methylbutyl)-(2*E*,6*Z*,8*E*)-decatrienamide, undeca-2E-en-8,10-dyinoic acid isobutylamide, *N*-isobutyl-2*E*,6*Z*-decenamide, and *N*-isobutyl-2E-decenamide [14]. Other components found in *H. longipes* root are squalene, β-sitosteryl palmitate, stigmasteryl palmitate, lupeyl acetate, lupeol, angelicoidenol, β-sitosterol, and stigmasterol [14]. In our previous study, a GC-MS analysis of the dichloromethane root extract led to the identification of 42 compounds including alkamides, carboxylic acids, aldehydes, esters, ethers, aromatic hydrocarbons, and terpenes (monoterpenes, sesquiterpenes and diterpenes) [15,16]. In another study, the volatile composition of *H. longipes* root was assessed by HS-SPME-GC-MS-TOF. This analysis showed that the most abundant volatiles where δ-3-carene (25.7%), α-terpinolene and 1,3,8-*p*-menthatriene (8.06% both), γ-cadinene (10.8%), and β-chamigrene (10.6%) [17].

On the other hand, a differential transcriptomic study performed by Buitimea-Cantúa et al. [18] showed that this plant expresses a broad range of genes involved in the biosynthesis o specialized metabolites. In the root, phenylpropanoid biosynthesis genes were highly expressed, while the leaves showed abundant expression of carotenoid-metabolic pathway genes [19].

*H. longipes* grows wild only in limited areas of the Sierra Gorda Biosphere Reserve in Mexico [20]. In some communities of the states of Queretaro and Guanajuato it is cultivated by rural inhabitants, who have used traditional cultivation methods [21]. Although this species is not considered to be threatened or in danger of extinction, its population has been significantly reduced due to excessive harvesting [1]. Understanding the metabolic response of *H. longipes* to the cultivation conditions and phenological stage will allow, on one hand, the establishment of suitable cultivation strategies for its preservation and propagation and, on the other hand, the proposal of the most appropriate time for harvesting when the concentration of bioactive compounds is increased. In this context, this study aimed to assess the influence of the phenological stage on the metabolome and the vasodilatory activity of *H. longipes* roots.

## 2. Results

### 2.1. Identification of Phenological Stages

**Figure 1:**
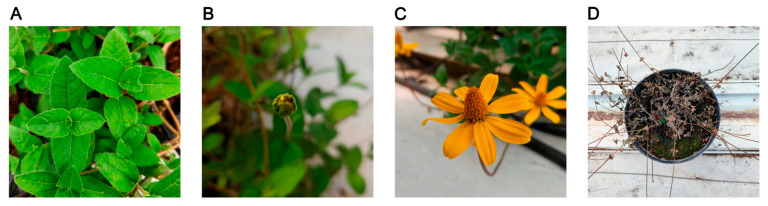
Phenological stages observed in *H. longipes* specimens acclimated under greenhouse conditions. (**A**) Vegetative stage (pre-flowering), (**B**) flowering stage (flower bud formation), (**C**) fruit-setting stage (achene and seed development), and (**D**) defoliation stage (partial or complete leaf loss).

In the vegetative stage, specimens were characterized by aerial shoots with nine or more unfolded leaves and a developed root (BBCH: stage 1–9). During flowering, plants exhibited visible floral organs with inflorescences clearly separated from the foliage leaves (stage 5–7). Specimens bearing achenes were grouped into the fruit-setting stage (stage 8–0), showing fruit-setting ripening with seeds at the margin of the flower head and on the underside of the green flower head. Finally, specimens with partial or complete leaf loss were classified as defoliated or senescent (stage 9–7) (Figure 1).

### 2.2. Morphological Parameters

**Figure 2:**
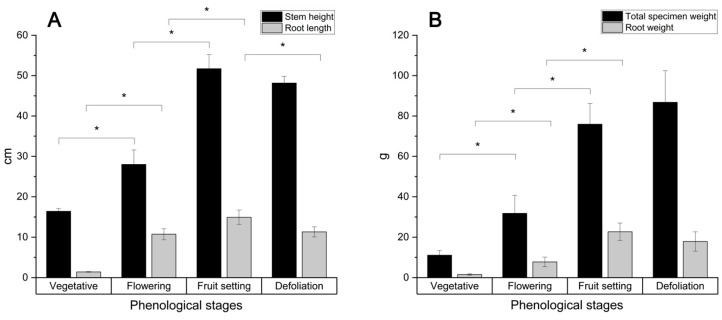
Morphological variables measured in specimens grown under greenhouse conditions at different phenological stages: (**A**) stem height and root length and (**B**) Total specimen and root weights. Data are expressed as mean ± SEM (*n* = 6). An asterisk (*) indicates a significant difference among phenological stages (*p* = 0.05). Statistical significance was assessed using one-way ANOVA followed by Tukey’s multiple comparisons test (*p* < 0.05).

The results of acclimatizing the specimens under greenhouse conditions suggest a significant increase in all variables as the phenological stages progressed. A gradual increase was observed in all parameters throughout development, except during the defoliation stage, where a slight decrease was recorded. The morphological data for each stage are shown in Figure 2.

Regarding stem height and root length, the fruit-setting and defoliation stages showed the greatest stem height and root length, although no significant difference was observed between them.

Concerning biomass, the same behavior observed in stem height and root length was found, with both increasing significantly as phenological stages progressed until fruit-setting. Specifically, the fruit-setting and defoliation stages showed total and root masses approximately 6.0 and 6.5 times greater, respectively, than the vegetative stage. Conversely, no significant differences were found in these variables during the fruit-setting and defoliation stages in the specimens. While significant differences were observed between the first three stages, the fruit-setting stage consistently showed the highest values for all morphological variables.

### 2.3. NMR Metabolomic Analysis

**Figure 3:**
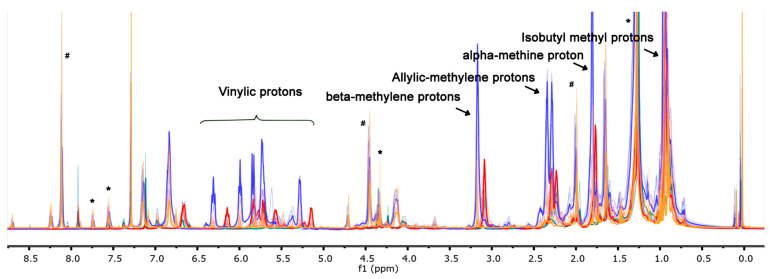
Representative ^1^H NMR spectra (700 MHz, 298 K, TMS 0.03% *v*/*v*, CDCl_3_) of dichloromethane extracts obtained from *H. longipes* roots at different phenological stages under greenhouse conditions: vegetative (yellow trace), flowering (blue trace), fruit-setting (red trace), and defoliation (green trace). The symbols (*) and (#) indicate the signals corresponding to di-*n*-butyl phthalate and polybutylene Terephthalate, respectively.

The ^1^H NMR spectra obtained from dichloromethane extracts of *H. longipes* roots grown under greenhouse conditions across all phenological stages revealed a complex metabolic profile predominantly characterized by signals associated with alkamide-type structures. Spectra acquired in CDCl_3_ at 700 MHz exhibited consistent resonance patterns across all samples, with notable variations in signal intensity in chemical shift regions associated with aliphatic chains (δ 0.8–2.5 ppm), olefinic protons (δ 5.0–6.5 ppm), and amide-related functional groups (δ 1.5–4.5 ppm) (Figure 3). These resonance patterns were mainly consistent with affinin, the major alkamide previously reported in *H. longipes* roots.

Furthermore, resonance signals inconsistent with the endogenous metabolic profile of *H. longipes* were identified. The observed spectral patterns were consistent with previous reports of plastic-derived contaminants [22]. These signals were assigned to di-*n*-butyl phthalate [23] (Figure S1A,C and Table S1) and polybutylene terephthalate, compounds widely reported as ubiquitous environmental contaminants (Figure S2A,C and Table S2) [24].

The presence of phthalates in plant-derived samples has been extensively documented and may result from multiple sources, including environmental exposure (e.g., contaminated soils, agricultural plastics such as mulching films or irrigation systems, and atmospheric deposition) as well as contact with polymer-based materials during routine handling and processing [22].

**Figure 4:**
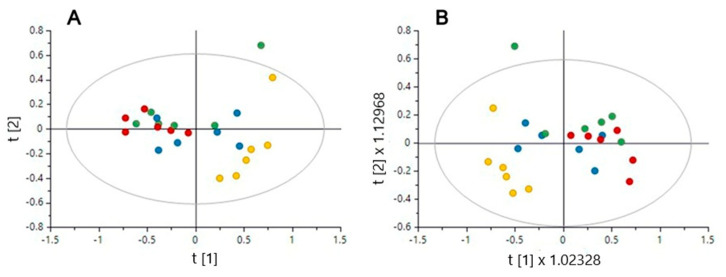
Multivariate analysis of dichloromethane extracts from *H. longipes* samples grown under greenhouse conditions at different phenological stages: (**A**) PCA score plot and (**B**) OPLS-DA score plot. Extracts were color-coded by phenological stage with six biological replicates per group: vegetative (yellow), flowering (blue), fruit-setting (red), and defoliation (green).

Exploratory principal component analysis (PCA) of the ^1^H NMR spectra revealed that nearly all samples were contained within the Hotelling’s T^2^ 95% confidence ellipse. Only one defoliation observation was identified as a potential outlier; however, no clear biological or experimental justification was found to support their exclusion; therefore, all samples were retained for further analysis. In this PCA, no distinct segregation of samples according to phenological stage was observed, although the vegetative sample were the only group showing partial separation from the rest. The model explained a substantial proportion of the variance (*R*^2^*X*(cum) = 0.935) and showed good predictive ability (***Q*^2^**(cum) = 0.837) using two components, indicating robust descriptive and predictive performance (Figure 4A).

The supervised OPLS-DA model provided improved, although moderate, discrimination of samples according to phenological stage (*R*^2^*X*(cum) = 0.805, *Q*^2^(cum) = 0.201). The effect of the signals assigned to phthalates was evaluated using parallel analyses with and without these spectral regions. The consistency between the resulting models, which maintained metrics with non-significant differences, allowed for the identification of changes in the chemical composition of the plants associated with the phenological stage. However, given that phthalate signals appeared consistently in the samples, it was decided to retain them in subsequent analyses, as they could represent environmental exposures present during plant development and be associated with the actual chemical profile. The score plot showed separation primarily along the first predictive latent variable t[1], which accounted for 41.5% of the explained variance. Vegetative samples were the only group showing some degree of separation, positioned toward the negative side of t[1]. In contrast, flowering, fruit-setting, and defoliation samples remained largely overlapping (Figure 4B). To better capture the discrete biochemical shifts associated with plant ontogeny, pairwise OPLS-DA models were prioritized over a global multiclass model. This approach facilitates the identification of stage-specific biomarkers by isolating the variance associated with individual phenological transitions (e.g., the onset of flowering), which might otherwise be obscured by the broader variance across the entire growth cycle.

**Figure 5:**
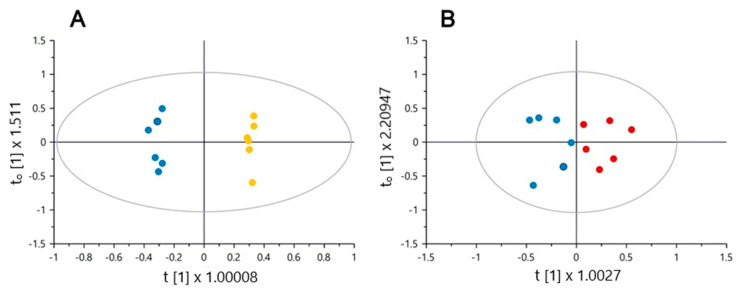
OPLS-DA score plots illustrating metabolic discrimination across phenological stages: (**A**) vegetative (VEG) vs. flowering (FWL) and (**B**) FWL vs. fruit-setting (FRS). Samples were color-coded according to phenological stage, with six biological replicates per group.

The OPLS-DA model constructed for the first two developmental stages (Figure 5A) showed high robustness, with an *R^2^X*(cum) of 0.880 and a *Q*^2^(cum) of 0.879, indicating excellent predictive performance. Further validation metrics are presented in Figure S3 and Table S3A,B. The variation explained by the model was distributed into the predictive component *t*[1] and two orthogonal components t*_o_*[1], t*_o_*[2]. During the vegetative stage, the loading profile indicated a predominance of long aliphatic chains, consistent with a metabolic composition enriched in fatty acid precursors and saturated structures. In contrast, the flowering stage exhibited a metabolic shift toward alkamide-associated signals, particularly in spectral regions where resonances corresponding to amide-containing structures are typically observed. Although signal overlap suggests that structurally related metabolites may also contribute, these spectral features support the increasing relevance of alkamides during reproductive development.

In contrast, the OPLS-DA model comparing the flowering and fruit-setting stages (Figure 5B) exhibited lower predictive capacity (*R*^2^*X*(cum) = 0.812, *Q^2^*(cum) = 0.451), suggesting a greater degree of metabolic similarity between these reproductive phases. Further validation metrics are presented in Figure S4 and Table S4A,B (Supplementary Materials). The model consisted of one predictive and one orthogonal component. Despite the limited discrimination, the loading profile revealed a sustained increase in signal intensities during the fruit-setting stage in regions commonly associated with alkamide resonances. The persistence and progressive intensification of these signals suggest that alkamides remain dominant contributors to the metabolic profile of *H. longipes* roots throughout reproductive development.

The final OPLS-DA analysis comparing the fruit-setting and defoliation stages did not generate a valid predictive model, indicating substantial metabolic similarity between these two developmental stages.

### 2.4. Purification and Structural Elucidation of the Major Alkamides Contained in the Dichloromethane Extract

Since the ^1^H NMR-based metabolomic analysis revealed a metabolic profile predominantly characterized by affinin-related resonances, while minor alkamides could not be individually resolved due to their low abundance and spectral overlap, a complementary phytochemical approach was undertaken to identify additional alkamide constituents present in the dichloromethane extract of *H. longipes* roots. Diagnostic resonances associated with the isobutyl moiety of affinin were clearly observed, including the terminal methyl groups at δ 0.96 ppm, the methine proton at δ 1.8–2.0 ppm, and the methylene group adjacent to the amide nitrogen (N–CH_2_) at δ 3.16 ppm, all characteristic of amide-containing structures. Additionally, the signal at δ 2.28 ppm was assigned to allylic protons within the unsaturated carbon chain. This interpretation was further supported by resonances in the unsaturated region (δ 5.16–8.32 ppm), particularly the signals at δ 5.72, 5.88, and 6.28 ppm, which are consistent with the conjugated triene system of affinin. Based on these observations, a preparative HPLC method was developed to purify the major alkamides present in the extract, followed by their structural elucidation using spectroscopic techniques.

**Figure 6:**
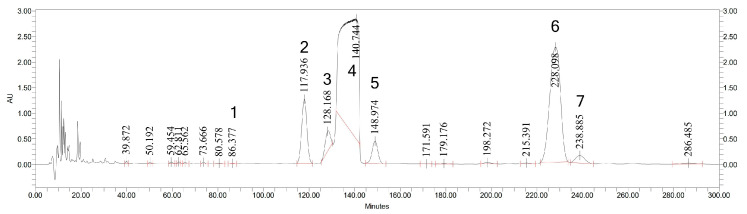
Preparative chromatogram of the alkamide-rich fraction obtained from the dichloromethane extract of *H. longipes* roots.

The dichloromethane extract prepared from the roots of *H. longipes* was subjected to column chromatography, yielding a 14 g fraction enriched in alkamides. Thin-layer chromatography revealed a single dark brown spot upon detection with ammonium ceric sulfate, indicative of nitrogen-containing compounds [25]. This alkamide-rich fraction was subsequently analyzed using a preparative HPLC-DAD method (Figure 6).

The preparative HPLC-DAD method enabled the isolation of the major alkamides present in the alkamide-rich fraction. Structural elucidation was carried out through detailed analysis of two-dimensional NMR experiments, including COSY, HSQC, and HMBC correlations. The experimental NMR spectra obtained in this study are provided in the Supplementary Materials. Molecular weights determined by mass spectrometry further supported and confirmed the proposed structures. Compound identification was consistent with previously reported data. Compound 1 was identified as *N*-isobutylundeca-2(*E*)-en-8,10-diynamide, obtained as a pale-yellow oil (Figure S5A–E and Table S5). Its isomer, compound 1′, was characterized as *N*-isobutylundeca-3(*E*)-en-8,10-diynamide (HPLC-DAD: λ_max_ 202, 217, 263 nm; RT 86.377 min; C_15_H_21_NO) (Figure S6A–C and Table S6). Compound 2, *N*-isobutyl-2(*E*),4(*Z*)-undecadiene-8,10-dynamide, was obtained as a pale-yellow oil (HPLC-DAD: λ_max_ 195, 258.3 nm; RT 117.936 min; C_15_H_19_NO) (Figure S7A–E and Table S7). The structural characterization of compound 3 was limited by the low availability of purified material (<2 mg); however, MS data indicated a molecular formula similar to that of affinin (HPLC-DAD: λ_max_ 210.2, 233.6 nm; RT 128.168 min; C_14_H_23_NO) (Figure S8A,B) [26]. Compound 4, *N*-isobutyl-2(*E*),6(*Z*),8(*E*)-decatrienamide (affinin or spilanthol), was obtained as a pale-yellow oil (HPLC-DAD: λ_max_ 231.3 nm; RT 140.744 min; C_14_H_23_NO) (Figure S9A–E and Table S8). Compound 5, also obtained as a pale-yellow oil, exhibited the same molecular formula (C_14_H_23_NO) as compounds 3 and 4. However, its UV–Vis profile (λ_max_ 198.5, 228.9 nm) and retention time (RT 148.97 min) differed from those of affinin, suggesting that it may correspond to a different isomeric form (Figure S10A–E and Table S9). Compound 6 was identified as *N*-(2-methylbutyl)-2(*E*),6(*Z*),8(*E*)-decatrienamide (homospilanthol), obtained as a pale-yellow oil (HPLC-DAD: λ_max_ 231.3 nm; RT 228.098 min; C_15_H_25_NO) (Figure S11A–E and Table S10). Finally, the characterization of compound 7 was also limited by its low yield (<2 mg) (HPLC-DAD: λ_max_ 198.5, 230.1 nm; RT 238.885 min).

Notably, based on an extensive review of the available literature, this study represents the first report of *N*-isobutylundeca-2(*E*)-en-8,10-diynamide (1), *N*-isobutylundeca-3(*E*)-en-8,10-diynamide (1′), and *N*-isobutyl-2(*E*),4(*Z*)-undecadiene-8,10-diynamide (2) in the roots of *H. longipes*.

### 2.5. Affinin Content

**Figure 7:**
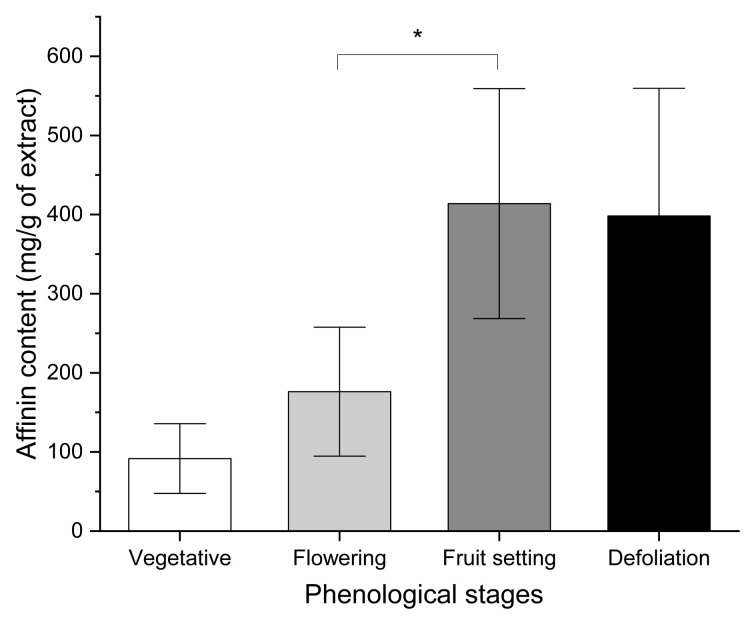
Effect of phenological stage on affinin concentration in the roots of *H. longipes*. Data are expressed as mean ± SEM (*n* = 6). An asterisk (*) denotes a significant difference among phenological stages. Statistical significance was assessed using one-way ANOVA followed by Tukey’s multiple comparisons test (*p* < 0.05).

Following the structural characterization of the major alkamides present in the dichloromethane extract, affinin quantification was performed using an HPLC-DAD method previously developed and standardized by our research group, whose analytical conditions are described in the Section 4 [27]. The quantification of affinin demonstrated that this compound is present from the earliest developmental stages of *H. longipes* and persists throughout its entire growth cycle. Notable variations in affinin concentration were observed across phenological stages, with the highest levels detected during the fruit-setting stage and the lowest during the vegetative stage, following a progressive increase along plant development. Importantly, these results are in strong agreement with and provide quantitative validation of the trends previously identified by ^1^H NMR analysis, reinforcing affinin as the most dynamically regulated and representative metabolite across the evaluated phenological stages (Figure 7).

**Figure 8:**
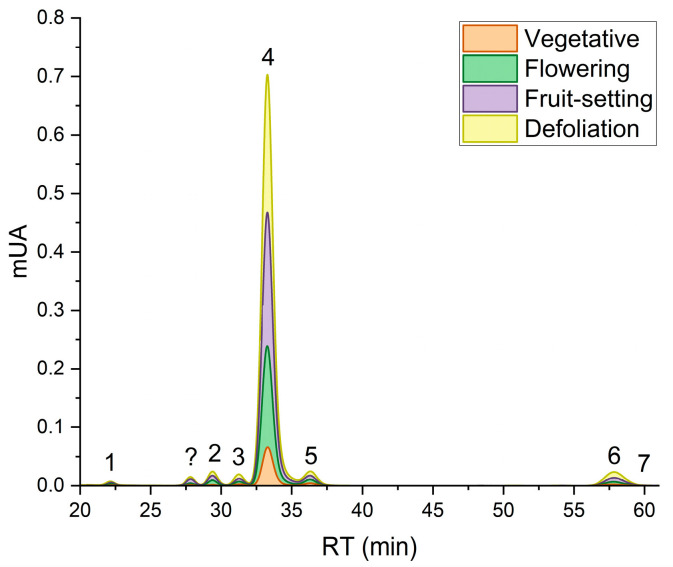
Representative HPLC-DAD chromatograms (λ = 236 nm) of alkamides detected in *H. longipes* roots at different phenological stages. Chromatograms are shown for illustrative purposes only; quantitative affinin determinations and statistical analyses are presented in Figure 7. Peaks: (1) *N*-isobutylundeca-2(*E*)-en-8,10-diynamide and *N*-isobutylundeca-3(*E*)-en-8,10-diynamide, (2) *N*-isobutyl-2(*E*),4(*Z*)-undecadiene-8,10-diynamide, (3) unknown, (4) affinin, (5) affinin isomer, (6) homospilanthol, and (7) unknown.

The phenological stage influenced not only affinin concentration but also the accumulation of minor alkamides, as evidenced by the representative chromatograms shown in Figure 8. The vegetative stage exhibited the lowest proportions of both affinin and related alkamides, whereas the fruit-setting and defoliation stages showed the highest signal intensities, although these minor components were not individually quantified. Notably, due to their low relative abundance and potential signal overlap, these alkamides could not be resolved or identified by ^1^H NMR analysis. Overall, these patterns suggest that the metabolic flux toward alkamide biosynthesis intensifies during the reproductive phase, predominantly favoring affinin accumulation.

### 2.6. Comparative Analyses of Chemical Profile of Dichloromethane Extracts from the Root of H. longipes at Different Phenological Stages, Using Gas Chromatography Coupled to Mass Spectrometry

The GC-MS analysis of the dichloromethane extracts from *H. longipes* roots revealed a complex matrix of semi-volatile compounds. The metabolic profile was predominantly composed of alkamides (14–41%) and lipid derivatives (11–36%) associated with the root cuticle. These chemical classes exhibited distinctive variations across the different phenological stages of the crop. A detailed list of the identified compounds, along with their respective biosynthetic classifications, is provided in Table S11A–D (Supplementary Materials).

Overall, a clear transition was observed from a profile dominated by structural components during early development toward one characterized by specialized secondary metabolites in later stages. This metabolic shift was primarily driven by the progressive accumulation of alkylamides, which displaced cuticular lipids as the major chemical group.

During the vegetative stage, 60.87% of the low-molecular-weight volatile compounds were identified. This period was predominantly characterized by lipid derivatives associated with the root cuticle (36.38% of the total area), followed by alkamides (14.96%). Among the latter, affinin and *N*-isobutylundeca-2(*E*)-en-8,10-diynamide were the major constituents. In lower proportions, lignans (3.95%), fatty acids (3.71%), and terpenes (1.64%) were also detected.

Upon transitioning to the flowering stage, the total identification percentage reached 52.82%. Although cuticular lipid derivatives remained the dominant group (28.33%), a substantial increase in the alkamide fraction (22.47%) was observed. At this point, the relative abundance of affinin rose to accompanied by the emergence of homospilanthol and hexadecanamide, suggesting the onset of a metabolic specialization toward defense or signaling mechanisms.

This metabolic shift consolidated during the fruit-setting, where alkylamides displaced cuticular lipids as the preponderant chemical group, representing 31.43% of the profile (compared to 11.95% for lipids). Affinin remained the primary compound, followed by a notable increase in *N*-isobutyl hexadecanamide and homospilanthol. Interestingly, the highest abundance of phytosterols was recorded during this stage, while lignans and terpenes maintained marginal contributions. Finally, the defoliation stage achieved the highest degree of chromatographic characterization (72.15% of the total area). Despite foliar senescence, root cuticular lipid derivatives showed a relative recovery (28.13%), whereas other groups, such as terpenes and phenylpropanoids, were detected at trace levels (<1%).

Taken together, our results evidence a marked biosynthetic divergence dependent on the phenological stage of *H. longipes*. While the vegetative stage prioritizes the synthesis of lipid derivatives likely linked to the reinforcement of structural barriers in the growing root the reproductive and senescence stages redirect the carbon flux toward alkylamide production. This balance between structural cuticular lipids and specialized metabolites (particularly affinin) suggests a programmed adaptive response, in which investment in chemical defense intensifies toward the final phases of the phenological cycle.

On the other hand, this analysis identified the three main alkamides previously purified and used as standards in the NMR-based metabolomic analyses of this study. Two additional alkamides not previously reported for this species were also detected, namely *N*-isobutyl hexadecanamide and hexadecanamide, both present in low abundance.

Furthermore, despite the qualitative and quantitative variability of metabolites across the different phenological stages, a persistent metabolic profile was observed throughout the plant’s life cycle. This pattern suggests the presence of a core metabolome associated with fundamental physiological functions that remains stable despite ontogenetic transitions (Table 1).

### 2.7. Enzymatic Determination

**Figure 9:**
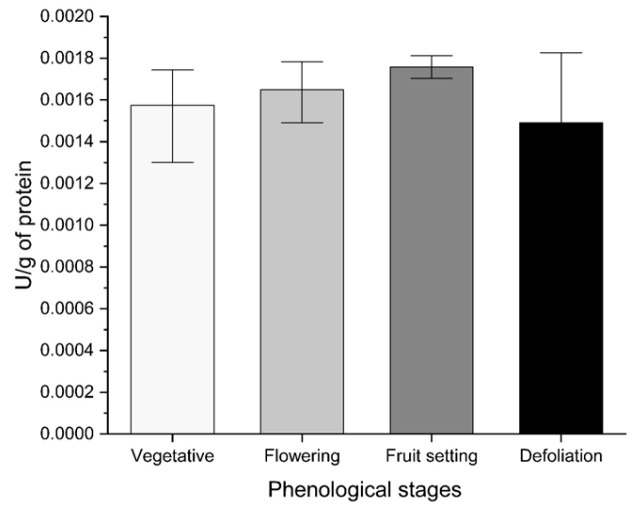
Effect of the phenological stage on valine decarboxylase (VDC) activity. Data are expressed as mean ± SEM (*n* = 6). No significant differences were detected among phenological stages (one-way ANOVA followed by Tukey’s multiple comparisons test (*p* < 0.05).

Although no significant differences in valine decarboxylase (VDC) enzymatic activity were observed among *H. longipes* root specimens at different phenological stages, the data suggest that the highest activity occurred during the fruit-setting stage, whereas the lowest was recorded during the defoliation stage. The corresponding data are presented in Figure 9.

### 2.8. Vasodilatory Effect on Isolated Rat Aorta

**Figure 10:**
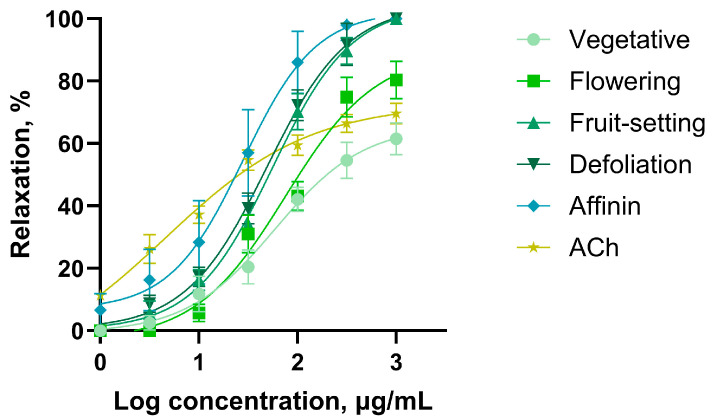
Concentration–response curves of the vasodilatory effect induced by root extracts of *H. longipes* specimens cultivated under greenhouse conditions at different phenological stages (*n* = 5). The responses were recorded in isolated rat aortic rings with functional endothelium.

The vasodilation assay results showed that dichloromethane root extracts from *H. longipes* specimens at different phenological stages induced concentration-dependent relaxation in aortic rings with functional endothelium, indicating consistent activity throughout plant development. The pharmacological evaluation revealed significant differences (*p* < 0.05) in the maximum effect (E_max_) and mean effective concentration (EC_50_) values among the different phenological stages. The corresponding concentration-response curves are presented in Figure 10, while the pharmacological parameters for each extract, acetylcholine (positive control), and affinin are summarized in Table 2.

The analysis of the vasodilator activity revealed significant variations in the efficacy of the extracts depending on the plant’s developmental stage. Extracts obtained during the vegetative stage presented the lowest maximum effect (E_max_), representing the lowest relaxation percentage achieved at the highest concentration tested, followed in ascending order by the flowering stage. In contrast, extracts from the fruit-setting and defoliation stages exhibited complete vasorelaxation (E_max_ = 100%), with no significant differences observed between them (*p* < 0.05). Regarding pharmacological potency (EC_50_), a similar behavior was observed across most phenological stages. The only statistically significant exception occurred during the flowering stage (*p* < 0.05), which showed a distinct potency compared to the other evaluated stages.

## 3. Discussion

Phenology constitutes a fundamental practical tool for harvest optimization, as visual stages serve as essential markers for identifying the precise point of chemical maturity. Characterizing metabolic variations as a function of phenological stage allows the determination of when maximum concentrations of compounds of interest are reached, enabling the implementation of rational production strategies that ensure both high yields and consistent phytochemical quality.

In this context, the present study provides the first formal description of the phenological development of *H. longipes* using the universal BBCH classification system, allowing precise and standardized identification of developmental stages. All stages, including defoliation, were successfully recognized, despite the inherent challenges associated with identifying vegetative structures under field conditions.

Consistent with plant developmental biology, morphological variables in *H. longipes* exhibited a progressive increase in biomass and plant height throughout ontogeny, reaching their absolute maximum values during the fruit-setting stage. Consequently, establishing this specific phase as the ideal harvest time guarantees the maximum recovery of root weight while securing the precise chemical profile required to achieve the desired therapeutic effect. This strategic timing provides the perfect equilibrium point for the development of phytopharmaceuticals and root-based products, as it simultaneously maximizes raw material yield and ensures high phytochemical consistency. Beyond structural growth, this transition toward physiological maturity reflects a critical metabolic shift from primary development to reproductive processes. During this stage, resource allocation shifts strategically to maximize root yield, establishing this organ as the primary source for the intensive biosynthesis and accumulation of specialized compounds. This physiological transition effectively overcomes the low biomass limitations that restrict the utility of earlier vegetative stages.

From a biomass valorization perspective, these findings are particularly relevant because *H. longipes* is commercially exploited primarily for its roots. The substantial increase in root biomass observed during the fruit-setting stage not only improves the recovery of plant material per cultivation cycle but also enhances the economic efficiency of phytopharmaceutical production by maximizing both biomass yield and the concentration of the principal bioactive metabolite. Consequently, the identification of this phenological stage provides a practical framework for optimizing harvesting strategies, promoting sustainable resource utilization, and supporting the industrial standardization of *H. longipes*-derived products.

In agreement with this developmental transition, affinin was detected from early phenological stages and persisted throughout the ontogenetic cycle, showing a progressive accumulation that culminated during the fruit-setting stage. This increase correlated positively with root biomass, suggesting growth-associated regulation. At the phytochemical level, ^1^H NMR analysis revealed a stable metabolic profile characterized by the consistent presence of affinin across all phenological stages, indicating that its biosynthesis is constitutive rather than temporally restricted. However, a clear quantitative modulation was observed, particularly in the increasing intensity of affinin signals during development. Minor alkamides were not detected by NMR, likely due to their low abundance and signal overlap, indicating that phenological variation primarily affects metabolite concentration rather than composition. Functionally, alkamides in the roots of this species have been associated with rhizosphere communication processes, including the regulation of pathogenic microorganisms [3,4,5,6,7,8,9,10,11,12,13,14,15,16,17,18,19,20,21,22,23,24,25,26,27,28,29] and the modulation of plant–environment interactions [30], as well as with the regulation of root development, suggesting their involvement in constitutive physiological pathways [31].

This interpretation was further supported by multivariate analysis, which revealed a pronounced metabolic differentiation between vegetative and reproductive stages. The transition from vegetative to reproductive stages represented the most pronounced metabolic shift, driven mainly by increased affinin levels, while later stages showed reduced discrimination, reflecting metabolic stabilization. These findings reinforce the role of affinin as the principal metabolic marker underlying phenology-dependent variability.

Complementary chromatographic analyses confirmed that, although minor alkamides are present and accumulate during reproductive stages, their low relative abundance limits their detection by NMR. Together, these results indicate that the metabolic flux toward alkamide biosynthesis intensifies during development, predominantly favoring affinin accumulation. A stage-dependent reorganization of the root metabolome was also evident throughout plant development. Early phenological stages were characterized by a metabolic profile enriched in lipid-derived compounds associated with structural and growth-related functions, whereas reproductive stages exhibited a progressive enrichment in alkamides. This transition suggests a shift from predominantly structural metabolism toward the accumulation of specialized metabolites involved in chemical defense, signaling, and ecological interactions. Such a pattern is consistent with the developmental reallocation of carbon and energetic resources commonly observed during plant maturation, in which physiological investment gradually shifts from biomass production to the synthesis and storage of bioactive secondary metabolites. The greater diversity and accumulation of alkamides observed during fruit-setting and defoliation stages further support the role of these compounds as important components of the adaptive chemical strategy of *H. longipes* roots.

In parallel, the purification and structural elucidation of major alkamides from the dichloromethane root extract established a robust chemical framework for subsequent phytochemical analyses. The use of preparative HPLC-DAD combined with NMR and mass spectrometry confirmed the presence of known compounds such as affinin and homospilanthol, supporting previous reports that identify alkamides as the dominant metabolites in this species [14]. Notably, three alkamides, *N*-isobutylundeca-2(*E*)-en-8,10-diynamide, *N*-isobutylundeca-3(*E*)-en-8,10-diynamide, and *N*-isobutyl-2(*E*),4(*Z*)-undecadiene-8,10-diynamide, were identified for the first time in *H. longipes* roots, expanding its known phytochemical profile and suggesting a more complex biosynthetic network. The structural diversity observed among these compounds, including isomeric forms, may have functional implications, as subtle variations in chemical structure can influence biological activity.

To quantitatively validate the metabolomic trends observed by ^1^H NMR analysis, affinin content was determined using an HPLC-DAD method previously developed and standardized by our research group. The quantification of affinin demonstrated that this compound is present from the earliest developmental stages of *H. longipes* and persists throughout its entire growth cycle. Notable variations in affinin concentration were observed across phenological stages, with the highest levels detected during the fruit-setting stage and the lowest during the vegetative stage, following a progressive increase along plant development. Importantly, these results are in strong agreement with and provide quantitative validation of the trends previously identified by ^1^H NMR analysis, reinforcing affinin as the most dynamically regulated and representative metabolite across the evaluated phenological stages.

In addition to alkamides, GC–MS analysis revealed a complementary metabolic framework composed of terpenes, lignans, and lipids, which contribute to both structural and defensive functions. This functional scaffold includes metabolites that play discrete and critical biological roles: terpenes and lignans reinforce chemical resistance against herbivory and pathogen attack, while the lipid fraction supports energy homeostasis and maintains the structural integrity required for primary metabolism. This coexistence of primary and specialized metabolites, supported by the complementarity of both analytical platforms, reflects a balanced metabolic strategy that sustains physiological stability while enabling environmental adaptation.

In particular, the ubiquitous presence of structural metabolites such as squalene and various phytosterols highlights the importance of cellular integrity in the species survival strategy. Squalene, in addition to being a key intermediate in triterpenoid biosynthesis, plays a fundamental role in modulating membrane fluidity and activating defense mechanisms [32]. Similarly, membrane stability under abiotic stress appears to be mediated by phytosterols such as stigmasterol and β- and γ-sitosterol isomers, which not only constitute essential components of lipid architecture but also function as modulators of adaptive responses to environmental conditions [33]. Finally, the detection of lupeone adds a relevant dimension to the defensive profile of the metabolome, as its documented antimicrobial activity [34] suggests a strategic metabolic investment toward sustained protection against pathogens throughout the life cycle [35], as well as a potential role in the modulation of root plasticity and growth [36].

In contrast to the progressive accumulation of biomass and metabolites, the enzymatic activity of VDC remained relatively stable across all phenological stages. This observation differs from the direct association between affinin concentration and VDC activity proposed by Parola-Contreras et al. [37], who suggested this enzyme as a potential biochemical marker for alkamide content. In the present study, the absence of such a correlation indicates that VDC activity alone is insufficient to reliably predict the biosynthetic flux of affinin.

Importantly, previous studies have reported that increases in VDC activity are primarily associated with stress-related physiological responses rather than developmental transitions. Parola-Contreras et al. [37] described enhanced enzyme activity under conditions linked to stress signaling, suggesting that its regulation may be more sensitive to environmental cues than to intrinsic ontogenetic progression. From this perspective, the relatively constant enzymatic activity observed here is consistent with plants developing under non-stressful conditions, where no strong induction of stress-responsive metabolic pathways would be expected.

This discrepancy highlights the complexity of affinin metabolic regulation and supports the notion that VDC activity does not constitute a limiting factor governing affinin accumulation during normal development. Consequently, and in agreement with Buitimea-Cantúa et al., factors such as the availability of decanoic acid, (2*E*,6*E*,8*E*)-decatrienoic acid, or other fatty acid-derived intermediates may represent the critical determinants controlling the final metabolic flux toward affinin biosynthesis.

Affinin-induced vasodilation appears to involve multiple signaling pathways, including NO/cGMP, CO/cGMP, H_2_S/K_ATP_, and PGI_2_/cAMP [7]. In addition, affinin has been shown to activate CB1 receptors as well as TRPA1 and TRPV1 channels, while simultaneously inhibiting L-type calcium channels [9]. The vasodilation effect of this compound has also been associated with the modulation of high-conductance calcium-activated potassium channels (BK_Ca_), which promotes membrane hyperpolarization and contributes to the reduction in vascular tone. Together, these pieces of evidence reinforce affinin as the pillar of the observed efficacy, whose potency is modulated by the dynamic interaction of the extract’s components.

However, the possibility that other minor alkamides contribute to the observed vasodilatory activity cannot be excluded, as these compounds occur within a complex chemical matrix in which affinin represents the predominant bioactive constituent. Although the vasodilatory properties of these individual minor alkamides have not yet been experimentally evaluated, they share important structural features with affinin, particularly the α,β-unsaturated amide moiety and the lipophilic hydrocarbon chain. These structural elements have been recognized as key determinants of biological activity in alkylamides, influencing their interaction with diverse molecular targets [38].

Interestingly, although the fruit-setting and subsequent phenological stages exhibited the highest affinin concentrations and maximum vasodilatory efficacies, the vegetative-stage extract displayed greater potency. This observation suggests that factors beyond affinin concentration alone may influence the pharmacological response. One possible explanation is the contribution of metabolites that are relatively more abundant during the vegetative stage. Among these, (+)-sesamin emerged as a discriminant metabolite in the metabolomic analysis and may be relevant given its reported ability to promote endothelium-dependent vasorelaxation through increased nitric oxide (NO) bioavailability. Previous studies have shown that sesamin can enhance endothelial nitric oxide synthase expression and reduce vascular oxidative stress through inhibition of NADPH oxidase activity [39]. Therefore, while affinin is likely the principal contributor to the overall vasodilatory effect, the greater potency observed in vegetative-stage extracts may reflect additive or synergistic interactions among multiple metabolites, including (+)-sesamin. Nevertheless, the present study was not designed to evaluate metabolite combinations, and therefore such interactions remain hypothetical and require dedicated pharmacological investigation in future studies.

Overall, the integration of phenological, metabolic, and pharmacological data demonstrates that *H. longipes* exhibits a strategy of qualitative metabolic stability combined with quantitative specialization. Affinin emerges as the most representative and dynamically regulated metabolite, whose accumulation is closely linked to developmental progression. Importantly, these findings indicate that the optimal harvest stage depends not only on maximizing compound yield but also on balancing biological potency, highlighting that peak phytochemical accumulation does not necessarily coincide with maximum pharmacological efficacy.

## 4. Materials and Methods

### 4.1. Plant Material

*H. longipes* specimens were collected in the locality of Beltrán (21°16′15.5856″ N, 100°03′11.4624″ W), Xichú, Guanajuato, Mexico, with the aim of obtaining compounds to be used as reference standards for metabolomics analyses. Voucher specimens were taxonomically identified and deposited in the Jerzy Rzedowski Herbarium, Faculty of Natural Sciences, Autonomous University of Queretaro (voucher number: QMEX00006951). For studies assessing the effect of the phenological stage on the alkamide profile of *H. longipes* roots, plants were grown under greenhouse conditions (Table S12A,B). Seeds were germinated in plug trays using soil collected from Xichú, Guanajuato, as substrate. Seedlings with fully developed true leaves were subsequently transplanted into grow bags and acclimated for three months (Table S13).

### 4.2. Growing Conditions

All specimens (*n* = 24) were transplanted into 7 kg white and black bellows bags. The plants were then transferred to a curved-roof greenhouse located on the Amazcala Campus of the Autonomous University of Querétaro (20°42′15.5″ N 100°15′34.2″ W). The substrate consisted of a 4:4:2 ratio of Peat moss^®^, compost, and sieved tezontle, respectively. The specimens were irrigated by drip irrigation using an automated system with Steiner solution. During the experiment, temperature and humidity data were recorded in the greenhouse using an Elitech-GSP-6 (Jiangsu Elitech Electronic Technology Co., Ltd., Xuzhou, Jiangsu, China) thermo-hygrometer. Soil nutritional parameters, soil characterization, water properties, and living conditions in the greenhouse and at the collection site were determined according to NOM-021-RENAC-2000 [40].

The four phenological stages were identified according to seasonal patterns and morphological descriptions previously reported by Cilia-López et al. [20]. Each main stage was further subdivided based on the criteria established in the extended BBCH scale [41].

### 4.3. Morphological Data

Six specimens (*n* = 6) were randomly collected in each phenological stage and morphological parameters, such as plant height, stem width, and root length were measured with a YOMYM^®^ OQ-40/40S laser distance meter (Shenzhen Jiuman Technology Co., Ltd., Shenzhen, China). Biomass (complete specimen and its root) was measured using an Ohaus^®^ triple arm™ series 700 mechanical scale (Ohaus Corporation, Parsippany, NJ, USA).

### 4.4. Preparation of Organic Extracts

The roots were shade-dried and then powdered using an IKA^®^ MF 10.5 mm electronic mill (IKA-Werke GmbH & Co. KG, Staufen, Germany). Subsequently, the plant material was subjected to an extraction process by maceration with reagent-grade dichloromethane (J.T. Baker, Avantor Performance Materials, LLC, Radnor, PA, USA). This procedure was performed in triplicate. The solvent was removed by rotary distillation under reduced pressure (BÜCHI Labortechnik AG, Flawil, Switzerland) until a dry extract was obtained.

### 4.5. ^1^H NMR Analysis of the Dichloromethane Extracts Obtained from the Roots of H. longipes Specimens Collected at Different Phenological Stages

#### 4.5.1. Sample Preparation

Extracts of roots obtained from *H. longipes* specimens in different phenological stages were dissolved in 700 μL of deuterated chloroform (99.8%, CDCl_3_, +0.03% *v*/*v* TMS, Cambridge Isotope Laboratories, Inc., Tewksbury, MA, USA) and transferred to a 5 mm × 7 in Norell SV-SUPER NMR tube (Norell, Inc., Morganton, NC, USA). NMR analyses spectra were recorded at a frequency of 700 MHz with a *z*-axis gradient TCI cryoprobe (5 mm) on an Avance II HD 700 NMR spectrometer (Buker, Billerica, MA, USA).

#### 4.5.2. ^1^H NMR Analysis

^1^H NMR measurements were recorded on a Bruker Avance III HD 700 MHz spectrometer equipped with a 5 mm *z*-axis gradient TCI cryoprobe (Buker, Billerica, MA, USA). ^1^H NMR spectra were acquired at 298 °K using standard pulse sequences. An exponential line broadening factor of 0.3 Hz was applied to the free induction decay (FID) prior to Fourier transformation. All ^1^H NMR spectra were automatically processed by phased-, baseline-corrected and referenced internally to the chemical shift in TMS at 0.00 ppm using the TopSpin software (version 3.5.6, Bruker Biospin, Rheinstetten, Germany).

#### 4.5.3. Multivariate Date Analysis

Data from each spectrum were reduced by generating homogeneous bins of 0.04 ppm over a chemical shift range of 0.23 to 11.33 ppm, excluding the solvent signal (7.23–7.33 ppm) through the Chenomx NMR Suite software (version 8.3, Chenomx Inc. Edmonton, AB, Canada). The ^1^H NMR data matrix was processed using Pareto scaling, and exploratory principal component analysis (PCA), and orthogonal projections to discriminants of latent structures (OPLS-DA) were performed using SIMCA software (version 18.1, Sartorius Stedim Data Analytic AB, Umeå, Sweden). 

### 4.6. Phytochemical Isolation and Purification of Alkamides from H. longipes Roots

A phytochemical study was performed to isolate and purify alkamides from the roots of *H. longipes*, which were used as standards for the metabolomic analysis of the extracts obtained from specimens of *H. longipes* in different phenological stages under greenhouse conditions.

#### Purification and Structural Elucidation of the Major Alkamides Contained in the Dichloromethane Extract

14 g of the dichloromethane extract from *H. longipes* roots was fractionated by open column chromatography. The stationary phase was silica gel Kiesegel 60, 100–230 mesh, 8 × 100 (Merk KGaA, Darmstadt, Germany) and the mobile phase started with *n*-hexane with the polarity being gradually increased until dichloromethane and subsequently with increasing volumes of ethyl acetate (J.T. Baker^®^). The process was monitored by thin-layer chromatography and fractions were grouped by chromatography similarity to yield 10 fractions. The alkamide-rich fraction was subjected to high-performance chromatography using a Waters 600E HPLC system (Waters Corporation, Milford, MA, USA) coupled to a Waters 2998 photodiode array detector (Waters Corporation, Milford, MA, USA). A Waters symmetryPrep™ C18 column (7.8 × 300 mm, 7 µm) was used as the stationary phase and acetonitrile with acidified water (1% acetic acid) 60:40 in isocratic mode was used as the mobile phase. The injection volume was 250 µL with a flow rate of 1 mL/min. UV detection was performed at a wavelength of 236 nm. HPLC separation resulted in the detection of six chromatographic peaks, which were dissolved in 700 μL of deuterated chloroform (99.8%, CDCl_3_, with 0.03% *v*/*v* TMS; Cambridge Isotope Laboratories, Inc., Tewksbury, MA, USA) and subjected to one-dimensional (^1^H, ^13^C) and two-dimensional (COSY, HSQC, and HMBC) NMR analyses. Spectra were recorded at 700 MHz using a *z*-axis gradient TCI cryoprobe (5 mm) on an Avance II HD 700 NMR spectrometer (Bruker, Billerica, MA, USA), with tetramethylsilane (TMS) as the internal standard.

### 4.7. Affinin Quantification

Affinin quantification in the extracts was carried out using a high-performance liquid chromatograph (Waters^®^ 600E) coupled to a photodiode array detector (Waters^®^ 2998). Separation was achieved on an Agilent^®^ ZORBAX Eclipse XDB-C8 column (5 µm, 4.6 × 150 mm, 120 Å) using acetonitrile and acidified water (1% acetic acid) (65:35, *v*/*v*) as the mobile phase under isocratic conditions. The injection volume was 20 µL, the flow rate was 1 mL/min, and detection was performed at 236 nm. The calibration curve was constructed using six concentrations of affinin standard (ChromaDex, Inc., Los Angeles, CA. USA) ranging from 15 to 90 µg/mL. All samples were analyzed in triplicate. This HPLC-DAD methodology was previously developed and analytically validated by our research group for the quantification of affinin in *H. longipes* preparations and was subsequently reported by Marrero-Morfa et al. [27]. A summary of the analytical validation parameters, including system suitability, linearity, accuracy, precision, limit of detection (LOD), limit of quantification (LOQ) and accuracy and repeatability of the method, is provided in the Supplementary Materials (Table S14).

### 4.8. GC-MS Analysis

The extracts and chromatographic peaks were analyzed by gas chromatography using an Agilent 7890A system equipped with a flame ionization detector (Agilent Technologies, Inc., Santa Clara, CA, USA) and an HP-5MS capillary column (60 m × 0.25 mm I.D.). The oven temperature was set at 150 °C for 5 min, increased at 5 °C/min to 230 °C, and held for 15 min. Helium was used as the carrier gas at a flow rate of 1 mL/min. Mass spectrometry analysis was performed using an Agilent 5975C EI quadrupole mass spectrometer at 70 eV, with ion source and quadrupole temperatures of 230 °C and 250 °C, respectively. Mass spectra were acquired in the range of 33 to 600 amu at 5.3 µS/s. A homologous series of n-alkanes was used as internal standards. Data processing was performed using MSD Agilent ChemStation software (version E.01.00.237, Agilent Technologies, Inc., Santa Clara, CA, USA) and compound identification was based on mass spectral comparison with the NIST^®^ v. 2.0 library.

### 4.9. Enzymatic Activity of Valine Descarboxylase

The enzymatic determination of VDC was carried out in the fresh root of each acclimated specimen and in its different phenological stages. Protein quantification by the Bradford method and enzymatic activity were measured using an UV-Vis spectrophotometer at 595 and 550 nm, respectively, employing the method reported by Cortez-Espinoza et al. [42] (Thermo Fisher Scientific, Waltham, MA, USA).

### 4.10. Pharmacological Evaluation of the Dichloromethane Extracts

Pharmacological evaluation was performed on male Wistar rats (*n* = 5) weighing 200–250 g. Animals were purchased from the Neurobiology Institute of the National Autonomous University of Mexico, Juriquilla Campus. During their stay, the animals were maintained under standard laboratory conditions with free access to water and food (Rodent LabDiet 5001, PMI Nutrition International, LLC, Richmond, IN, USA). Animal handling was in accordance with NOM-062-ZOO-1999 and the recommendations of the International Council for Laboratory Animal Science (ICLAS) [43]. The study protocol received approval from the Bioethics Committee of the Faculty of Chemistry, Autonomous University of Querétaro, Mexico (approval ID: CBQ25/005b). For the isolated rat aorta assay, the animals were sacrificed by decapitation under inhalational anesthesia with isoflurane. This assay was performed as described by Ibarra-Alvarado et al. [44]. The ascending aorta was cleaned of blood and connective tissue with cold Krebs–Henseleit solution. The aorta was cut into 4 mm rings, which were mounted in tissue isolation chambers with Krebs–Henseleit solution under physiological conditions (37 °C, pH 7.4) and constant oxygenation with O_2_/CO_2_ (95:5%). The tissue was contracted with 1 μM L-phenylephrine. Changes in tension produced by the extracts were recorded on a Grass FT03 force transducer coupled to a Grass 7D polygraph. Data are expressed as percentage of vasodilation based on the contraction generated by L-phenylephrine.

### 4.11. Statistical Analysis

Results are presented as mean ± standard error of the mean (SEM) obtained from three replicates of six independent samples (*n* = 6). Data visualization was performed using OriginPro software (version 10.1, OriginLab Corporation, Northampton, MA, USA) except for pharmacological analyses, which were plotted using GraphPad Prism software (version 8.01, GraphPad software, Boston, MA, USA) Statistical significance was evaluated by one-way ANOVA followed by Tukey’s post hoc test. The pharmacological parameters were determined directly from the non-linear regression of the concentration-response curves. The half-maximal effective concentration (EC_50_) was calculated as the concentration required to induce 50% of the maximum relaxation. The maximum effect (E_max_) was defined as the highest percentage of vasodilation achieved at the maximum concentration evaluated, where a lower E_max_ indicates lesser efficacy or a reduced ceiling effect among the evaluated phenological stages.

## 5. Conclusions

The findings of this study demonstrate a clear association between the phenological stage, the metabolomic profile, and the vasodilatory activity of *H. longipes* roots. The fruit-setting stage represents the optimal condition for maximizing both biomass production and affinin accumulation, which coincided with high vasodilatory efficacy. In addition, the identification of three minor alkamides, *N*-isobutylundeca-2(*E*)-en-8,10-diynamide, *N*-isobutylundeca-3(*E*)-en-8,10-diynamide, and *N*-isobutyl-2(*E*),4(*Z*)-undecadiene-8,10-diynamide, reported for the first time in this species, expands the known phytochemical profile of *H. longipes* and suggests a more complex biosynthetic network than previously recognized.

The greater potency observed in vegetative-stage extracts, despite their lower affinin content, indicates that factors beyond affinin concentration alone may contribute to the pharmacological response. Metabolomic analyses identified differential metabolites among phenological stages, including affinin and (+)-sesamin, which may represent relevant candidates for future pharmacological investigation. However, the present study does not establish a causal relationship between individual metabolites, metabolite interactions, and vasodilatory activity. Likewise, the absence of a direct relationship between valine decarboxylase activity and affinin accumulation suggests that affinin biosynthesis is regulated by multiple factors and cannot be explained solely by this enzymatic marker.

Overall, these results provide a scientific basis for selecting harvest stages according to specific production goals and support the use of metabolomic approaches for the standardization of *H. longipes*-derived phytopharmaceuticals. Future studies involving isolated compounds and defined metabolite combinations will be necessary to elucidate the specific contribution of individual metabolites and their potential interactions to the observed biological activity.

## Figures and Tables

**Table 1:** Non-polar compounds identified by GC-MS across phenological stages.

Compounds	Vegetative	Flowering	Fruit-Setting	Defoliation
n-eicosane	0.0006%	0.1598%	4.2717%	0.2276%
Squalene	1.3554%	0.0%	0.1037%	0.0582%
(+)-sesamin	2.5974%	1.4199%	1.9082%	0.3214%
Stigmasterol	0.0%	0.0827%	0.5176%	0.7819%
-gamma/beta.-sitosterol	0.0102%	0.0%	1.0354%	0.2581%

**Table 2:** Vasodilatory effect induced by *H. longipes* root extracts on rat aorta precontracted with L-Phe with functional endothelium.

Phenological Stages	E_max_ (%)	EC_50_ (μg/mL)
Vegetative	65.13 ± 0.25 *^#^	58.11 ± 3.99
Flowering	80.41 ± 1.71 *^#^	99.8 ± 2.23
Fruit-setting	100 ± 0	56.72 ± 1.43
Defoliation	100 ± 0	49.33 ± 4.54
Ach	67.88 ±1.43	9.30 ± 1.14
Affinin	100 ± 3.10	27.38 ± 1.20

Data are expressed as mean ± SEM (*n* = 5). Asterisks (*) and pound signs (#) indicate significant differences in E_max_ and EC_50_ values, respectively. Significant differences were determined by one-way ANOVA followed by Tukey’s multiple comparisons test (*p* < 0.05).

## Data Availability

Dataset available on request from the authors.

## Supplementary Materials

The following supporting information can be downloaded at https://www.mdpi.com/article/10.3390/molecules31122114/s1, Figures S1A–S2C: HSQC and COSY NMR spectra and spectroscopic data (Tables S1 and S2) of the phthalates identified in the spectra; Figures S3 and S4: Statistical validation plots of OPLS-DA models (Figures S3A–S4B), variance tables (Tables S3A–S4B), and classification errors; Figures S5A–S11E: HSQC and COSY NMR spectra, mass spectra, and spectroscopic data of purified alkamides (Tables S5–S10); Tables S11A–S11D: Chemical profiles of non-polar compounds from dichloromethane extracts across different phenological stages; Tables S12A,B: Climate records of temperature and relative humidity inside the greenhouse; Table S13: Analysis of the substrate utilized in the greenhouse; Table S14: Analytical validation parameters and acceptance criteria for the HPLC-DAD quantification method of affinin.

## Abbreviations

The following abbreviations are used in this manuscript:

ACh	Acetylcholine
BBCH	Biologische Bundesanstalt, Bundessortenamt und CHemiche industrie
BKCa	high-conductance calcium-activated potassium channels
CDCI_3_	Deuterated chloroform
COSY	Correlation Spectroscopy Nuclear Magnetic Resonance
DEF	Defoliation stage
EC_50_	Half-maximal effective concentration
E_max_	Maximum effect
FLW	Flowering stage
FRS	Fruit-setting stage
GC-MS	Gas chromatography-coupled to mass spectrometry
HMBC	Heteronuclear Multiple Bond Correlation
HPLC-DAD	High-Performance liquid chromatography with diode array detection
HSQC	Heteronuclear Single Quantum Coherence
L-Phe	L-Phenylephrine
NMR	Nuclear Magnetic Resonance
NO	Nitric oxide
OPLS-DA	Orthogonal Projections to Latent Structures Discriminant Analysis
PCA	Principal Component Analysis
*Q*^2^(cum)	Cumulative predicted variation
*R*^2^*X*(cum)	Cumulative fraction of *X* variation explained
RMSEE	Root mean square error of stimation
RMSEcv	Root mean square error of cross-validation
SEM	Standard error of the mean
TMS	Tetramethyl silane
VDC	Valine decarboxylase
VEG	Vegetative stage

## References

[1] García-Chávez A., Ramírez Chávez E., Molina-Torres J. (2004). The genus *Heliopsis* (Heliantheaae; Asteraceae) in Mexico and the alkamides stored in its roots. Acta Bot. Mex..

[2] Gutiérrez-Lugo M.T., Barrientos-Benítez T., Luna B., Ramírez-Gama R.M., Bye R., Linares E., Mata R. (1996). Antimicrobial and cytotoxic activities of some crude drug extract from Mexican Medicinal Plants. Phytomedicine.

[3] Molina-Torres J., Salaza-Cabrera C.J., Armenta-Salinas C., Ramírez-Chávez E. (2004). Fungistatic and Bacteriostatic Activities of Alkamides from *Heliopsis longipes* Roots: Affinin and Reduced Amides. J. Agric. Food Chem..

[4] Hernández I., Márquez L., Martínez I., Dieguez R., Delporte C., Prieto S., Molina-Torres J., Garrido G. (2009). Anti-inflammatory effects of ethanolic extract and alkamides-derived from *Heliopsis longipes* roots. J. Etnofarmacol..

[5] Cariño-Torres R., Gayosso-De-Lucio J.A., Ortiz M.I., Sánchez-Gutiérrez M., García-Reyna P.B., Cilia-López V.G., Pérez-Hernández N., Moreno E., Ponce-Monter H. (2010). Antinociceptive, genotoxic and histopathological study of *Heliopsis longipes* S.F. Blacke in mice. J. Etnofarmacol..

[6] Cilia-López V.G., Juárez-Flores B.I., Aguirre-Rivera J.R., Reyes-Agüero J.A. (2010). Analgesic activity of *Heliopsis longipes* and its effect on the nervous system. Pharm. Biol..

[7] Castro-Ruiz E., Rojas-Molina A., Luna-Vázquez F.J., Rivero-Cruz F., García-Gasca T., Ibarra-Alvarado C. (2017). Affinin (spilanthol), Isolated from *Heliopsis longipes*, Induces Vasodilation via Activation of Gasotransmitters and Prostacyclin Sginaling Pathways. Int. J. Mol. Sci..

[8] Luz-Martínez B., Marrero-Morfa D., Luna-Vázquez F.J., Rojas-Molina A., Ibarra-Alvarado C. (2024). Affinin, isolated from *Heliopsis longipes*, Induces an Antihypertensive Effect That Involver CB1 Cannabinoid Receptors and TRPA1 and TRIV1 Channel Activation. Planta Med..

[9] Valencia-Guzman C.G., Castro-Ruiz J.E., García-Gasca T., Rojas-Molina A., Romo-Mancillas A., Luna-Vázquez F.J., Rojas-Molina J.I., Ibarra-Alvarado C. (2021). Endothelial TRP channels and cannabinoid receptors are involved in affinin-induced vasodilation. Fitoterapia.

[10] Barbosa A.F., de Carvalho M.G., Smith R.E., Sabaa-Srur A.U.O. (2016). Spilanthol: Occurrence, extractions, chemistry and biological activities. Rev. Bras. Farm..

[11] Rondanelli M., Fossari F., Vecchio V., Braschi V., Riva A., Allegrini P., Petrangolini G., Iannello G., Favila M.A., Peroni G. (2020). *Acmella olaraceae* for pain management. Fitoterapia.

[12] Jakupovic J., Schuster A., Chau-Thi T.V., Bohlmann F., Dominguez X.A. (1988). Guaianolides and homoditerpenes from *Heliopsis helianthoides*. Phytochemistry.

[13] Hajdu S., Haskó J., Krizbai I.A., Dezso C. (2014). Evaluation of Lignans from *Heliopsis helianthoides* var. Scabra for Their Potential Antimestastatic Effects in the Brain. J. Nat. Prod..

[14] López-Martínez S., Aguilar-Guadarrama A.B., Yolanda Ríos M. (2011). Minor alkamides from *Heliopsis longipes* S.F. Blake (Asteraceae) fresh roots. Phytochem. Lett..

[15] Ruiz-Castillo G.V. (2021). Caracterización Química del Extracto de Diclorometano de la Raíz de *Heliopsis longipes* y Evaluación del Efecto Vasodilatador de los Compuestos Mayoritarios Presentes en el Extracto. Master’s Thesis.

[16] Marrero-Morfa D., Luz-Martínez B.A., Luna-Vázquez F.J., Quirino-Barreda C.T., Rojas-Molina I., García-Servín M., Vázquez-Landaverde P.A., Ruiz-Castillo V., Ibarra-Alvarado C., Rojas-Molina A. (2025). Antihypertensive Effect of a Self-Microemulsifying System Obtained from and Ethanolic Extract of *Heliopsis longipes* Roots in Spontaneously and L-NAME-Induced Hypertensive Rats. Molecules.

[17] Aguilar M.I., Castillo N.E., Alvarado-López C., Duarte-Lisci G., Ríos-Gómez R., Rios M.Y. (2016). HPLC Determination of the Alkamide Affinin in Fresh and Dry Roots of *Heliopsis longies (*Asteraceae) and HS-SPME-GC-MS-TOF Analysis of Volatile Components. Food Anal. Methods.

[18] Buitimea-Cantúa G.V., Marsch-Martinez N., Ríos-Chavez P., Méndez-Bravo A., Molina-Torres J. (2020). Global gene expression analyses of the aljamide-producing plant *Heliopsis longipes* supports a polyketide synthase-mediated biosynthesis pathway. PeerJ.

[19] Buitimea-Cantúa G.V., Molina-Torres J. (2021). De novo transcriptome sequencing, assembly and characterization of *Heliopsis longipes* roots vs. Leaves to discover putative gene involver in specialized metabolitoes biosynthesis. Plant Omics J..

[20] Cilia-López V.G., Aguirre-Rivera J.R., Espinosa-Reyes G., Flores-Cano J.A., Reyes-Agüero J.A., Juárez-Flores B.I. (2013). Distribution of *Heliopsis longipes* (Heliantheae: Asteraceae), and Endemic resource from Central Mexico. Polibotánica.

[21] Cilia-López V.G., Aguirre-Rivera J.G., Reyes-Agüero J.A., Juárez-Flores B.I. (2008). Etnobotánica de *Heliopsis longipes* (Asteraceae: Heliantheae). Bol. Soc. Bot. Méx..

[22] Li X., Wang Q., Jian N., Lv H., Liang C., Yang H., Yao X., Wang J. (2022). Occurrence, source, ecological risk, and mitigation of phthalate (PAEs) in agricultural soils and the environment: A review. Environ. Res..

[23] Frankhauser-Noti A., Grob K. (2007). Bank problems in trace analysis diethylhexyl and dibutylphtalate: Investigation of the sources, tips and tricks. Anal. Chim. Acta.

[24] Zhou J., Jiang Z., Wang Z., Zhang J., Li J., Li Y., Zhang J., Chen P., Gu Q. (2013). Synthesis and characterization of triblock copolymer PLA-b-PBT-b-PLA and its effect on the crystallization of PLA. RSC Adv..

[25] Secretaria de Salud (2021). Farmacopea de los Estados Unidos Mexicanos.

[26] Alperth F., Feistrietzer T., Huber M., Kunert O., Bucar F. (2024). Natural Deep Eutectic Solvents for the Extraction of Spilanthol from *Acmella olaraceae* (L.) R.K. Jansen. Molecules.

[27] Marrero-Morfa D., Ibarra-Alvarado C., Luna-Vázquez F.J., Estévez M., Miranda-Ledesma E., Rojas-Molina A., Quirino-Barreda C.T. (2023). Self-microemulsifying system of an ethanolic extract of *Heliopsis longipes* root for enhanced solubility and release of affinin. AAPS Open.

[28] Molina-Torres J., García-Chávez A., Ramírez-Chávez E. (1999). Antimicrobial properties of alkamides present in flavouring plants traditionally used in Mesoamerica: Affinin and capsaicin. J. Ethnopharmacol..

[29] Mendez-Bravo A., Calderón-Vázquez C., Ibarra-Laclette E., Raya-González J., Ramírez-Chávez E., Molina-Torres J., Guevara-García A.A., López-Bucio J., Herrera-Estrella L. (2011). Alkamides Activate Jasmonic Acid Biosynthesis and Signaling Phatways and Confer Resistance to *Botrytis cinerea* in *Arabidopsis thaliana*. PLoS ONE.

[30] Ramírez-Chávez E., López-Bucio J., Herrera-Estrella L., Molina-Torres J. (2004). Alkamides Isolated from Plants Promote Growth and Alter Root Development in Arabidopsis. Plant Physiol..

[31] Woelkart K., Bauer R. (2007). The role of alkamides as an active principle of Echinacea. Planta Med..

[32] Thimmapa R., Geisler K., Louveau T., O’maille P., Osbourn A. (2014). Triterpene Biosynthesis in Plants. Annu Rev. Plant Biol..

[33] Schaller H. (2003). The role of sterols in plant growth and development. Prog. Lipid Res..

[34] Ngoufack Tagousop C., Feugap Tsamo D., Nguetse Dongmo A.J., Harakat D., Voutquenne-Nazabadioko L., Tamokou J.D., Nhnokam D. (2025). Chemical constituents from Ethyl acetate extract of *Graptohyllum glandulosum* Turrill and New semi-synthetic derivate with antimicrobial activities. Adv. Biol. Chem..

[35] Dong H., Qi X. (2025). Biosynthesis of triterpenoids in plants. Pathways, regulation and biological functions. Curr. Opin. Plant Biol..

[36] Bai Y., Fernández-Calvo P., Ritter A., Huang A.C., Morales-Herrera S., Bicalho K., Karady M., Pauwels L., Buyst D., Njo M. (2021). Modulation of *Arabidopsis* root growth by specialized triterpenes. New Phytol..

[37] Parola-Contreras I., Tovar-Perez E.G., Rojas-Molina A., Luna-Vázquez F.J., Torres-Pacheco I., Ocampo-Velazquez R.V., Guevara-González R.G. (2020). Changes in affinin contents in *Heliopsis longipes* (chilcuague) after a controlled elicitation strategy under greenhouse conditions. Ind. Crop. Prod..

[38] Elufioye T., Habtemariam S., Adjare A. (2020). Chemistry and Pharmacology of Alkylamides from Natural Origin. Rev. Bras. Farm..

[39] Nakano D., Kwak C.J., Fujii K., Ikemura K., Satake A., Ohkita M., Takaoka M., Ono T., Nakai M., Tomimori N. (2006). Sesamin metabolites induce an endothelial nitric oxide-dependent vasorelaxation through their antioxidative property-independent mechanism: Possible involvement of the metabolites in the antihypertensive effect of sesamin. J. Pharmacol. Exp. Ther..

[40] (2002). Which Establishes the Specifications for Fertility, Salinity, and Classification of Soils, Study, Samples and Analysis.

[41] Meier U. (2001). Growth Stages of Mono- and Dicotyledonous Plants: BBCH Monograph.

[42] Cortez-Espinosa N., Aviña-Verduzco J.A., Ramírez-Chávez E., Molina-Torres J., Ríos-Chávez P. (2011). Valine and Phenylalanine as Precursors in the Biosynthesis of Alkamides in *Acmella radicans*. Nat. Prod. Commun..

[43] (2001). Technical Specifications for the Production, Care and Use of Laboratory Animals.

[44] Ibarra-Alvarado C., Rojas A., Mendoza S., Bah M., Gutiérrez D.M., Hernández-Sandoval L., Martínez M. (2010). Vasoactive and antioxidant activities of plants used in Mexican traditional medicine for the treatment of cardiovascular diseases. Pharm. Biol..

