# Supplementary material for: Metabolomic Analysis of Heliopsis longipes Roots Across Phenological Stages
Source: Molecules. 2026 Jun 16;31(12):2114. doi: 10.3390/molecules31122114 (PMC13305561; doi:10.3390/molecules31122114)
Supplement: Supplementary file 1 [file molecules-31-02114-s001.zip › molecules-4338944-supplementary.pdf]

Article

Metabolomic analysis of *Heliopsis longipes* roots across phenological stages

Supplementary Materials

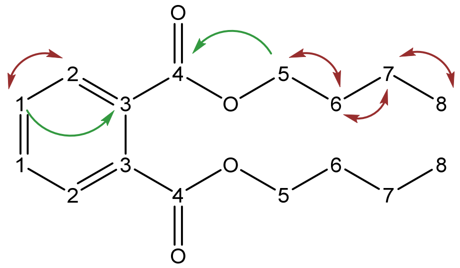

^{1}H NMR ($CDCl_3$ with 0.03% v/v TMS, 700 MHz): dH. 7.52 (dd, 3.29, 5.66), 7.71 (dd, 3.32, 5.71), 4.31 (t, 6.75), 1.72 (m), 1.44 (h, 7.41), 0.96 (t, 7.41). ^{13}C NMR ($CDCl_3$, 175 MHz): dC 131.11, 128.94, 130.82, 167.63, 65.27, 30.71, 19.31, 13.84.

Figure S1A. di-*n*-butyl phthalate

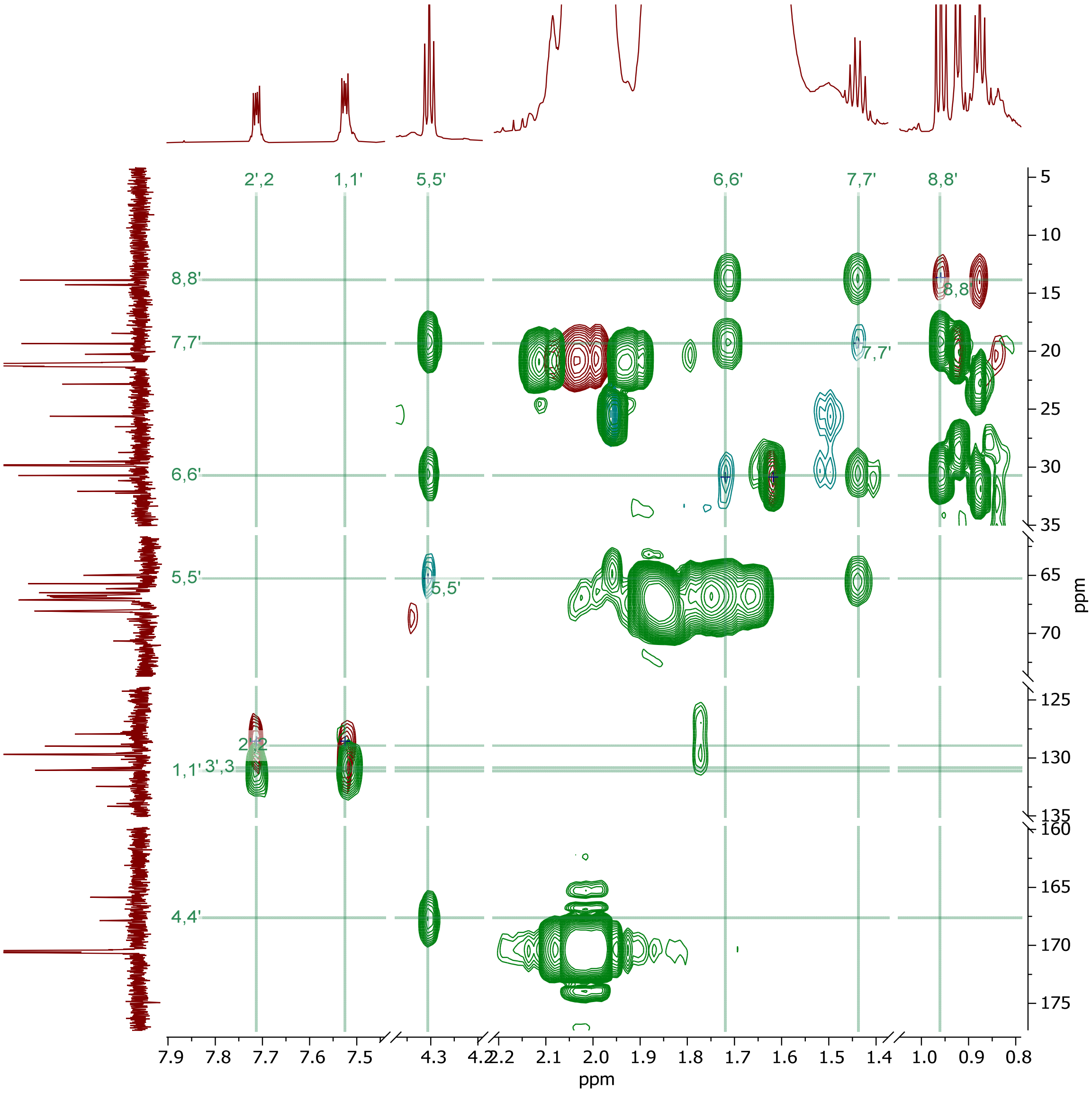

Figure S1B. ed-HSQC (red - blue) and HMBC (green) spectra (700 MHz, 300 K, $CDCl_3$).

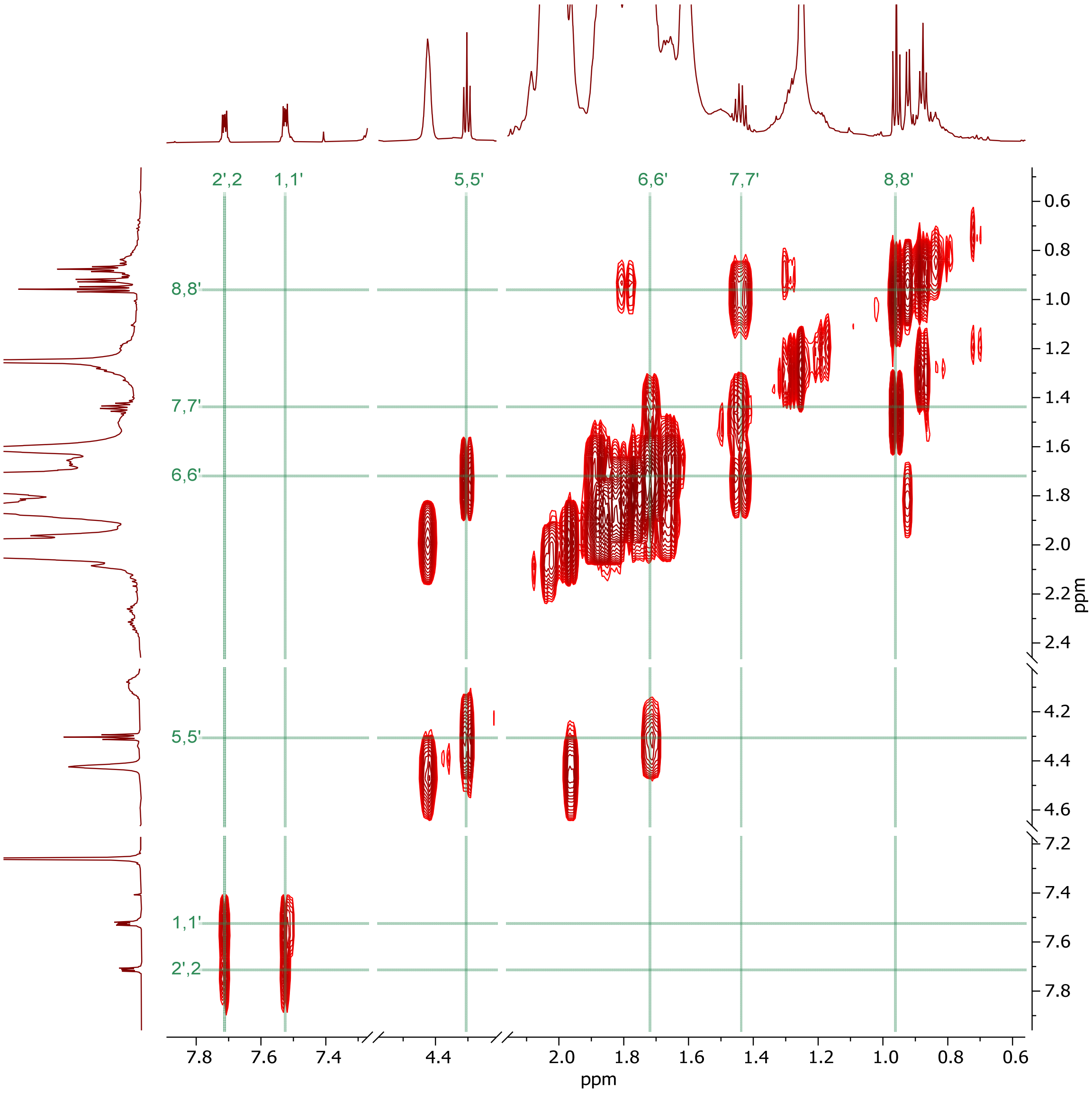

Figure S1C. COSY spectra (700 MHz, 300 K, $CDCl_3$).

Table S1. Spectroscopic data: d_H, d_C, HMBC and COSY (300 K, $CDCl_3$).

Assignation	δH/ppm (Multiplicity, J/Hz)	δC/ppm	HMBC (H→C)	COSY (H→H)
1	7.52 (dd, 3.29, 5.66)	131.11	C-2, 3	H-2
2,	7.71 (dd, 3.32, 5.71)	128.94	C-1, 3	H-1
3	-	130.82	-	-
4	-	167.63	-	-
5	4.31 (t, 6.75)	65.27	C-4, 6, 7	H-6
6	1.72 (m)	30.71	C-7, 8	H-5, 7

7	1.44 (h, 7.41)	19.31	C-5, 6, 8	H-6, 8
8	0.96 (t, 7.41)	13.84	C-6, 7	H-7

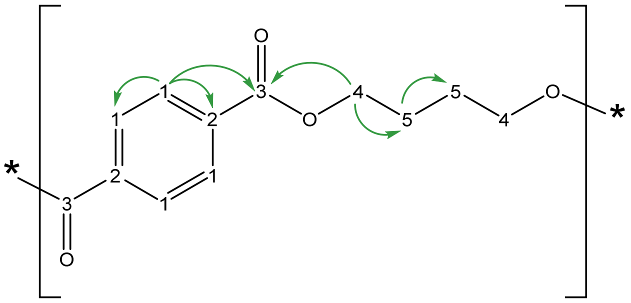

^{1}H NMR ($CDCl_3$ with 0.03% v/v TMS, 700 MHz): δ_H. 8.08 (bs), 4.42 (bs), 1.96 (bs). ^{13}C NMR ($CDCl_3$, 175 MHz): δ_C 129.74, 133.95, 165.69, 64.90, 25.68.

Figure S2A. Polybutylene terephthalate

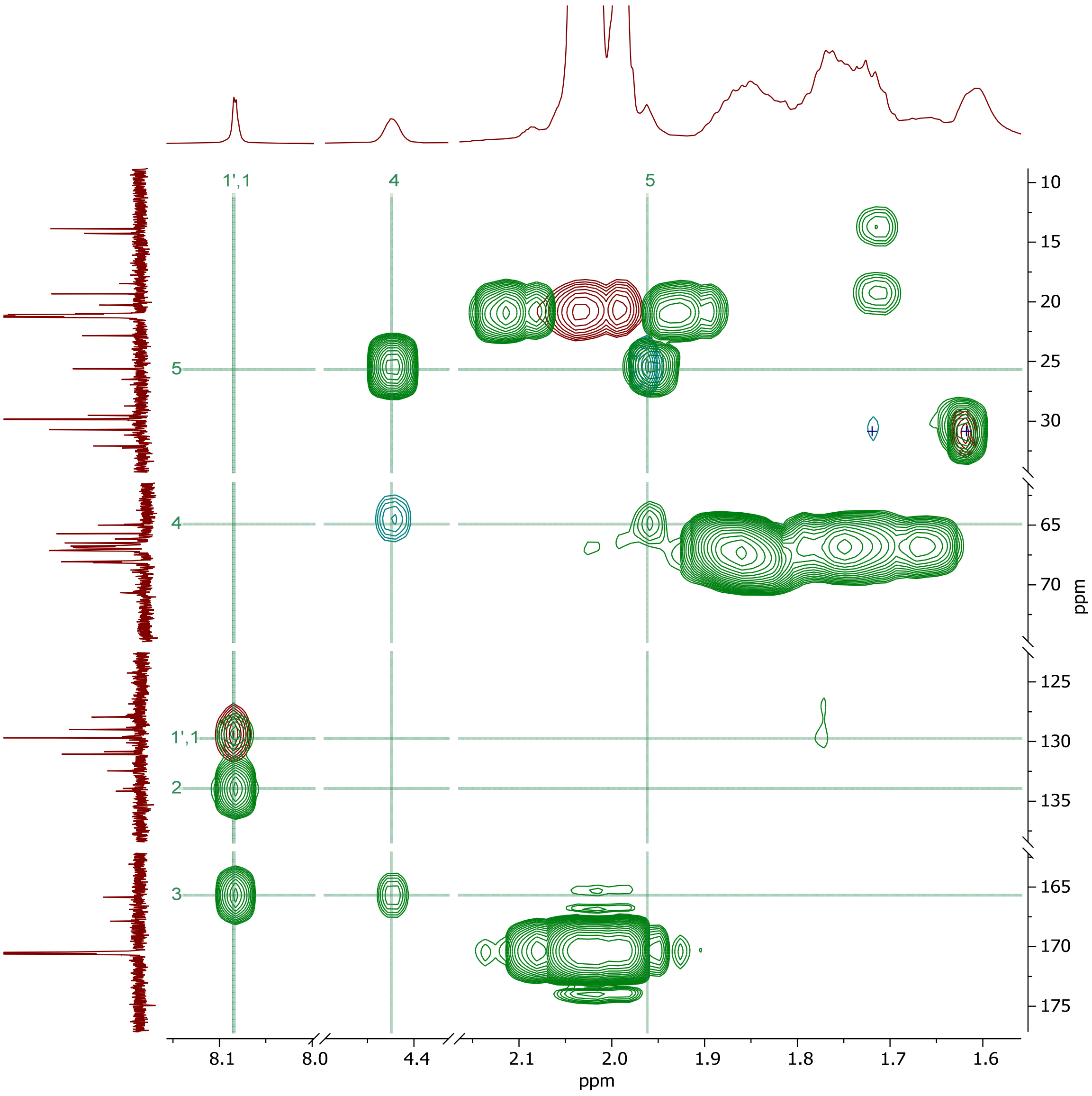

Figure S2B. ed-HSQC (red - blue) and HMBC (green) spectra (700 MHz, 300 K, $CDCl_3$).

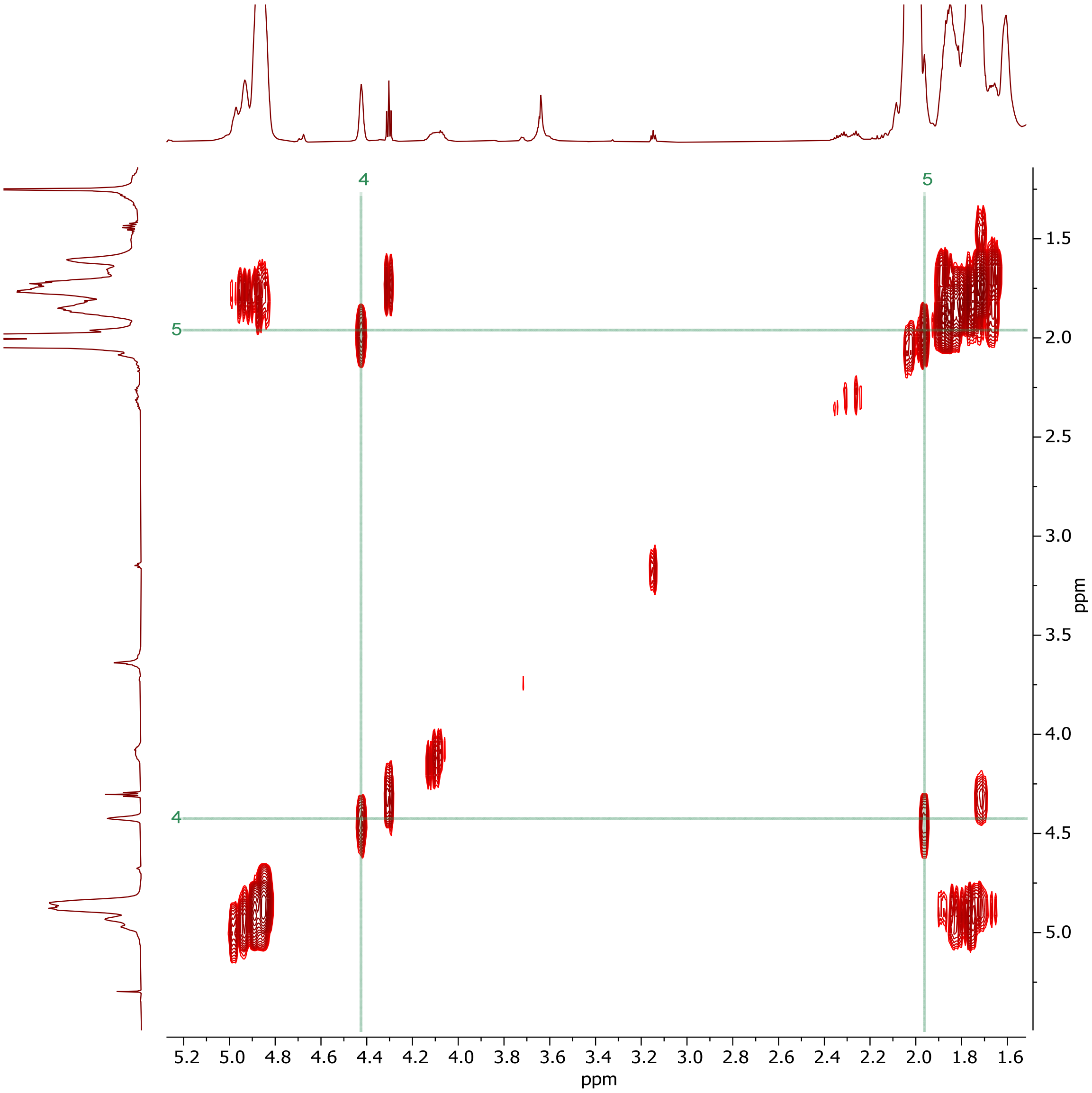

Figure S2C. COSY spectra (700 MHz, 300 K, $CDCl_3$).

Table S2. Spectroscopic data: d_H, d_C, HMBC and COSY (300 K, $CDCl_3$).

Assignation	δH/ppm (Multiplicity, J/Hz)	δC/ppm	HMBC<br>(H→C)	COSY<br>(H→H)
1	8.08 (bs)	129.74	C-1, 2, 3	-
2,	-	133.95	-	-
3	-	165.69	-	-
4	4.42 (bs)	64.90	C-3, 5	H-4
5	1.96 (bs)	25.68	C-4, 5	H-5

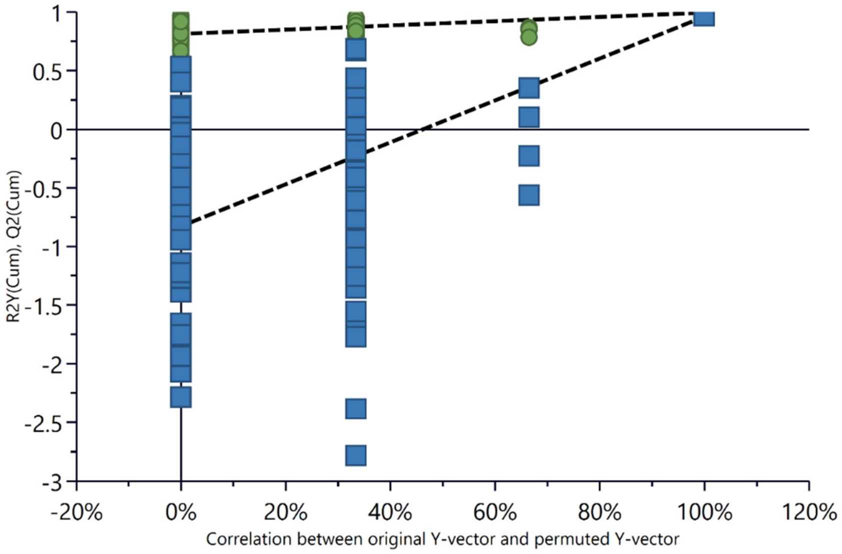

Figure S3. Permutation test plot for the OPLS-DA model classifying the extract samples according to the four phenological stages.

Table S3A. CV-ANOVA results for the OPLS-DA model classifying the extract samples according to the four phenological stages.

M7	SS	DF	MS	F	p	SD
Total corr.	11	11	1			1
Regression	10.563	6	1.760	20.135	0.002	1.326
Residual	0.437	5	0.087			0.295

Table S3B. Misclassification Table for the OPLS-DA model classifying the extract samples according to the four phenological stages.

	Members	Correct	B	A	No class (YPred <= 0)
B	6	100%	6	0	0
A	6	100%	0	6	0
No class	12		12	0	0
Total	24	100%	18	6	0
Fisher's prob.	0.0011				

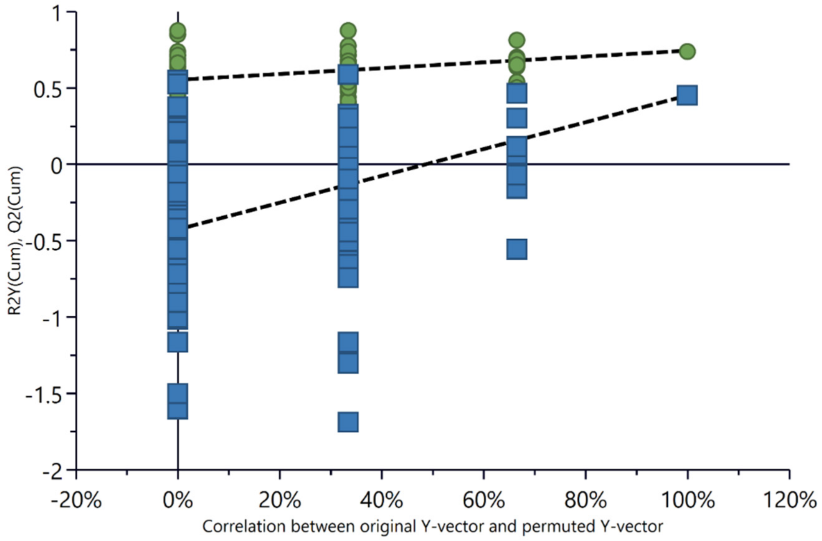

Figure S4. Permutation test plot for the OPLS-DA model classifying the extract samples according to the flowering and fruit-setting stages.

Table S4A. CV-ANOVA results for the OPLS-DA model classifying the extract samples according to the flowering and fruit-setting stages

M8	SS	DF	MS	F	p	SD
Total corr.	21	11	1			1
Regression	14.967	4	3.741	4.34	0.044	1.11
Residual	6.033	7	0.862			0.92

Table S4B. Misclassification Table for the OPLS-DA model classifying the extract samples according to the flowering and fruit-setting stages.

M8	Members	Correct	B	C	No class (YPred <= 0)
B	6	100%	6	0	0
C	6	100%	0	6	0
No class	12		9	3	0
Total	24	100%	15	9	0
Fisher's prob.	0.0011				

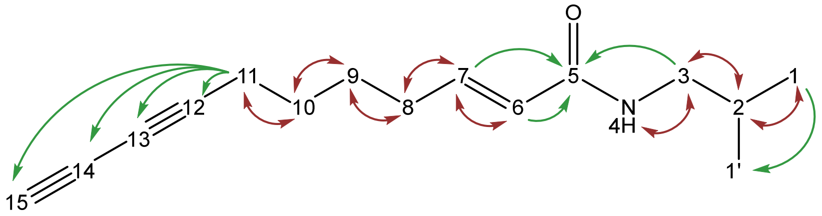

^{1}H NMR ($CDCl_3$ with 0.03% v/v TMS, 700 MHz): d_H 0.92 (d, 6.71, H1), 1.78 (m, H2), 3.15 (dd, 6.86, 6.09, H3), 5.43 (bs, H4), 5.79 (t, 1.59, H6), 6.81 (t, 6.88, H7), 2.20 (m, H8), 1.58 (m, H9), 1.58 (m, H10), 2.28 (td, 6.99, 1.29, H11), 1.96 (t, 1.18, H15).^{13}C NMR ($CDCl_3$, 175 MHz): d_C 20.25, 28.79, 46.98, 166.33, 124.21, 143.91, 31.60, 27.46, 27.41, 19.05, 77.85, 68.48, 68.59, 65.16.

Figure S5A. Compound 1. *N*-isobutylundeca-2(*E*)-en-8,10-diynamide.

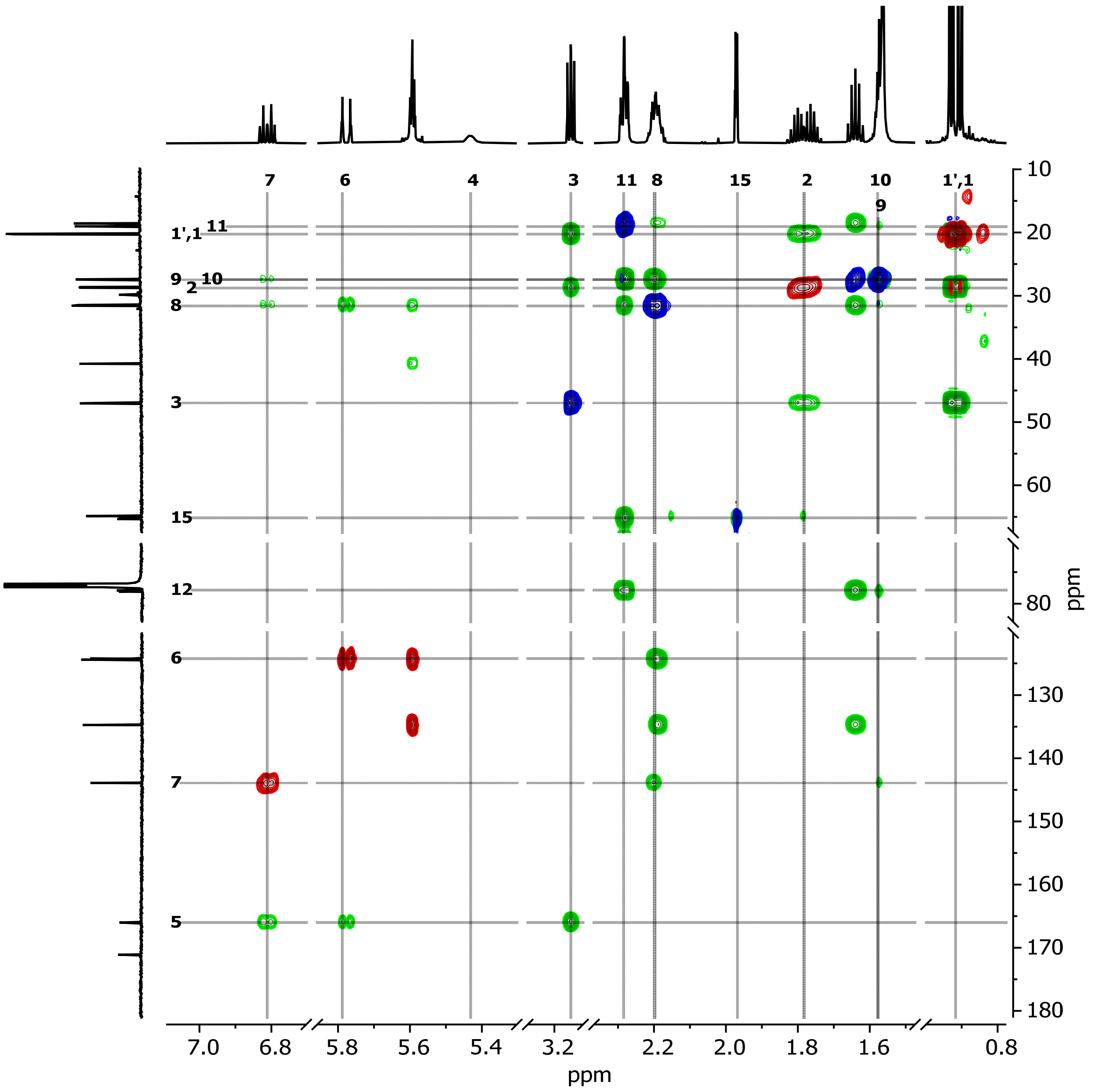

Figure S5B. ed-HSQC (red-blue) and HMBC (green) spectra (700 MHz, 300K, $CDCl_3$)

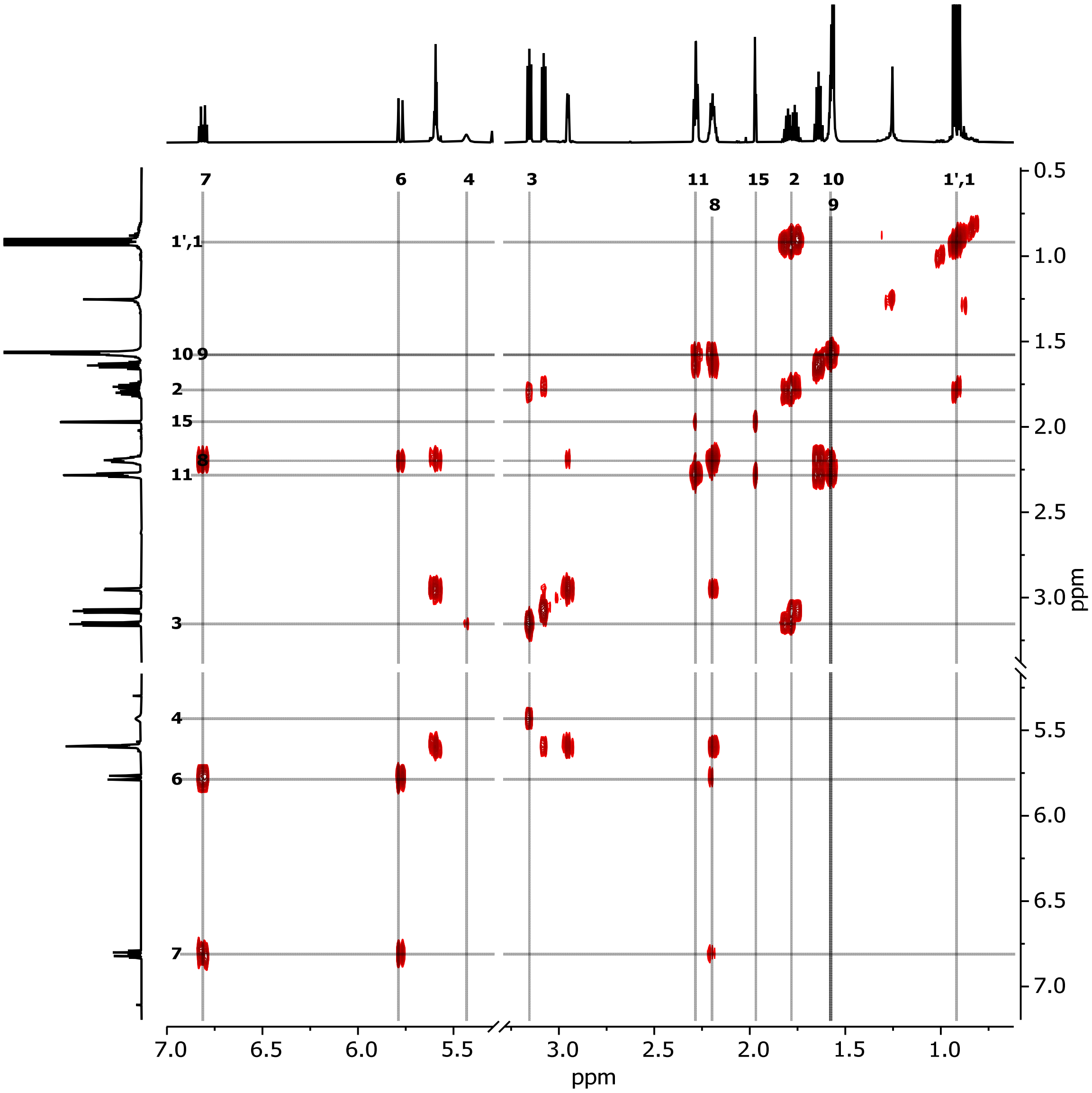

Figure S5C. COSY spectra (700 MHz, 300 K, $CDCl_3$).

Table S5. Spectroscopic data: d_H, d_C, HMBC and COSY (300 K, $CDCl_3$).

Assignation	δH/ppm (Multiplicity, J/Hz)	δC/ppm	HMBC (H→C)	COSY (H→H)
1	0.92 (d, 6.71)	20.25	C-1, 2, 3	H-2
2	1.78 (m)	28.79	C-1, 3	H-1, 3
3	3.15 (dd, 6.86, 6.09)	46.98	C-1, 2, 5	H-4
4	5.43 (bs)	-	-	H-3
5	-	166.33	-	-

6	5.79 (t, 1.59)	124.21	C-5, 8	H-7
7	6.81 (t, 6.88)	143.91	C-5, 6, 8, 9	H-6, 8
8	2.20 (m)	31.60	C-6, 7, 9, 10	H-7, 9
9	1.58 (m)	27.46	C-7, 8, 10, 11	H-8, 10
10	1.58 (m)	27.41	C-8, 9, 11, 12	H-9, 11
11	2.28 (td, 6.99, 1.29)	19.05	C-9, 10, 12, 13, 14, 15	H-10
12	-	77.85	-	-
13	-	68.48	-	-
14	-	68.59	-	-
15	1.96 (t, 1.18)	65.16	-	-

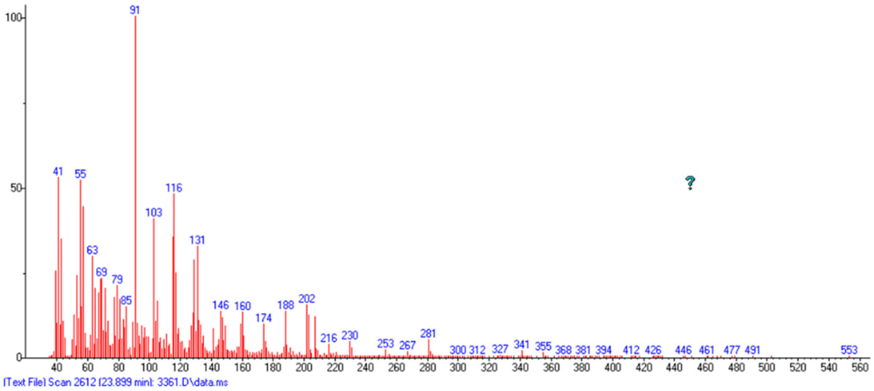

Figure S5D. Mass spectrum (EI, 70 eV) of compound 1 (*m/z* 33–600).

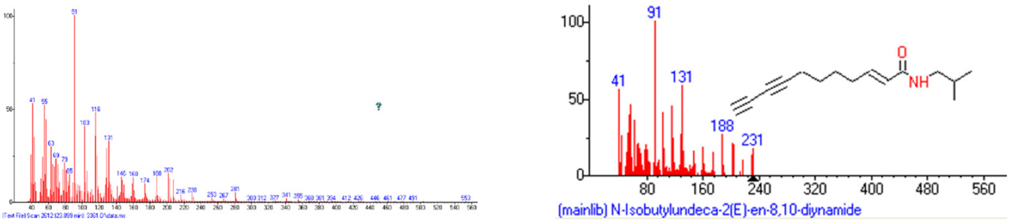

Figure S5E. Comparison of the experimental mass spectrum of compound 1 (A) with the NIST23 library reference for *N*-isobutylundeca-2(*E*)-en-8,10-diynamide (B).

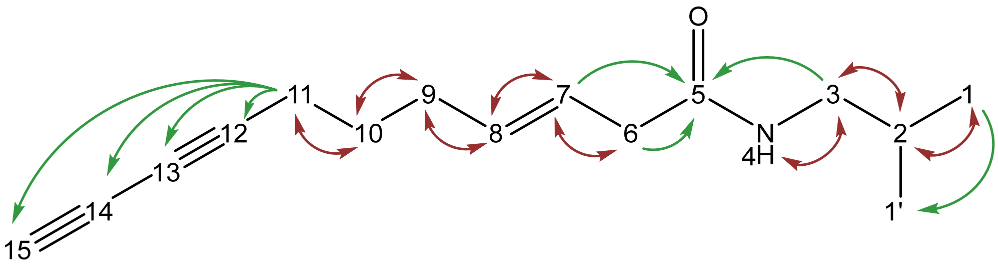

^{1}H NMR ($CDCl_3$ with 0.03% v/v TMS, 700 MHz): d_H 0.92 (d, 6.71, H1), 1.78 (m, H2), 3.15 (dd, 6.86, 6.09, H3), 5.43 (bs, H4), 2.96 (m, H6), 5.59 (m, H7), 5.59 (m, H8), 2.19 (m, H9), 1.64 (m, H10), 2.28 (td, 6.99, 1.29, H11), 1.97 (t, 1.18, H15). ^{13}C NMR ($CDCl_3$, 175 MHz): d_C 20.25, 28.79, 46.98, 166.33, 40.68, 123.76, 134.52, 32.15, 27.45, 18.84, 77.85, 68.48, 68.59, 65.16.

Figure S6A. Compound 1´: *N*-isobutylundeca-3(*E*)-en-8,10-diynamide.

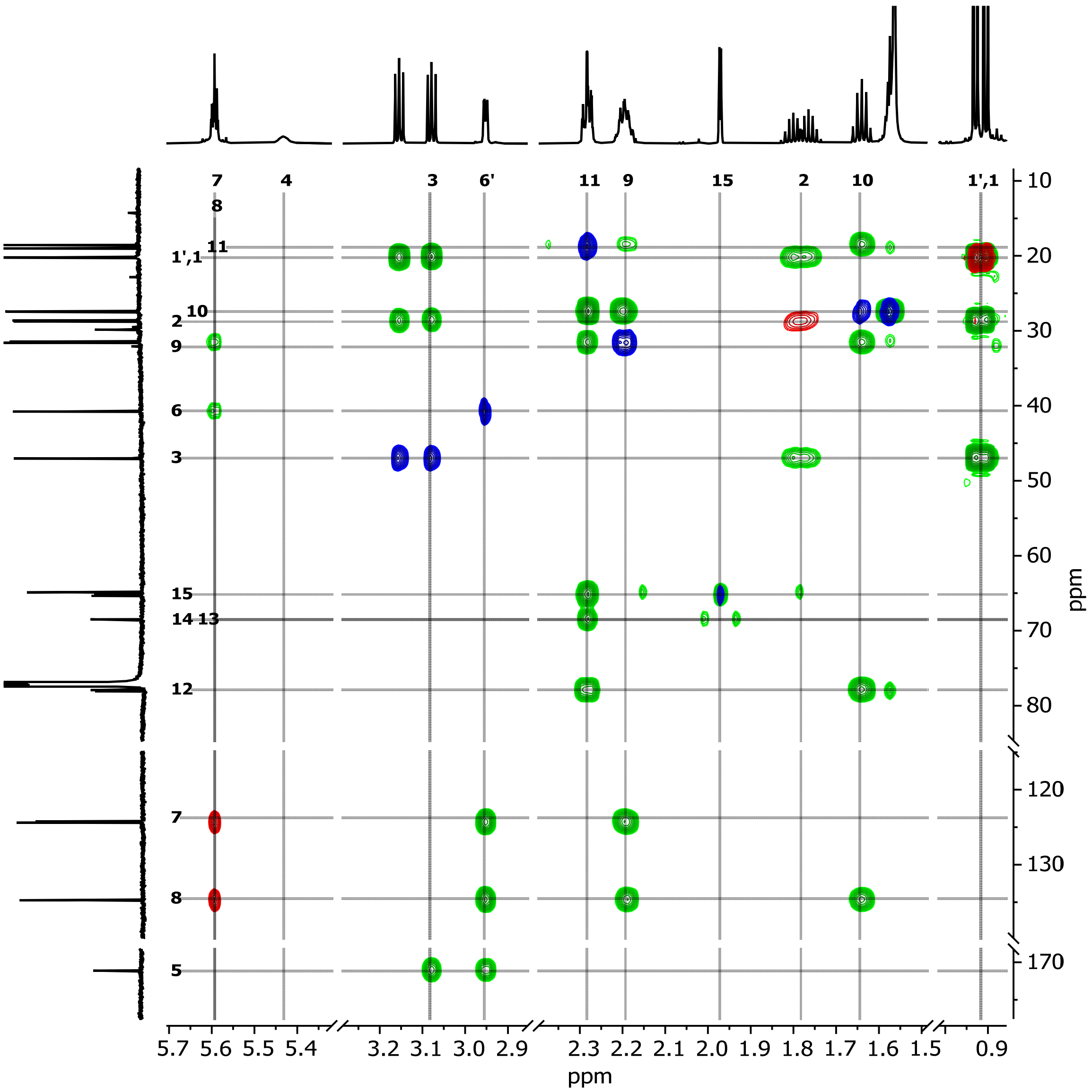

Figure S6B. ed-HSQC (red - blue) and HMBC (green) spectra (700 MHz, 300 K, $CDCl_3$).

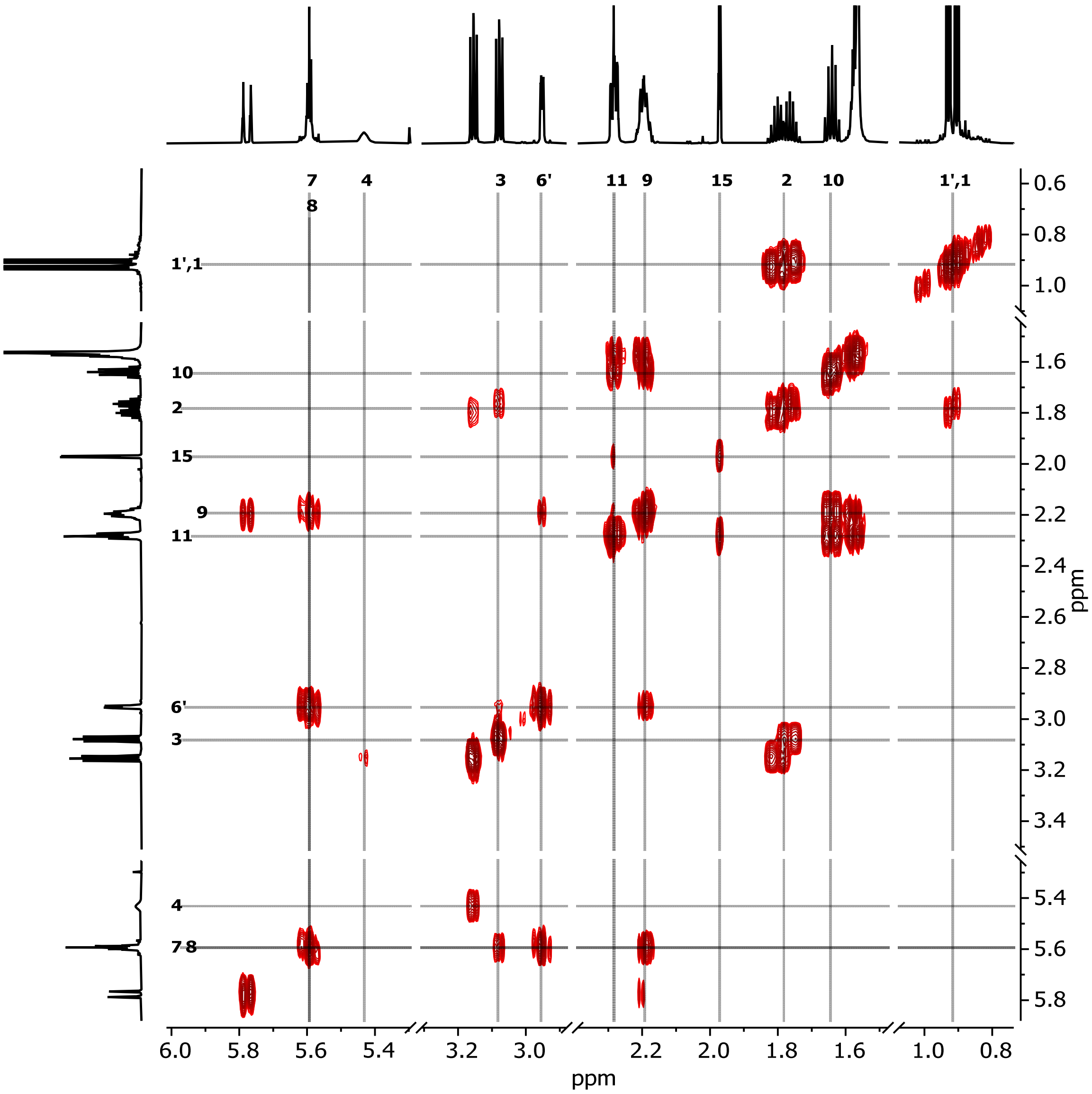

Figure S6C. COSY spectra (700 MHz, 300 K, $CDCl_3$).

Table S6. Spectroscopic data: d_H, d_C, HMBC and COSY (300 K, $CDCl_3$).

Assignation	δH/ppm (Multiplicity, J/Hz)	δC/ppm	HMBC (H→C)	COSY (H→H)
1	0.92 (d, 6.71)	20.25	C-1, 2, 3	H-2
2	1.78 (m)	28.79	C-1, 3	H-1, 3
3	3.15 (dd, 6.86, 6.09)	46.98	C-1, 2, 5	H-4
4	5.43 (bs)	-	-	H-3
5	-	166.33	-	-

6	2.96 (m)	40.68	C-5, 7, 8	H-7
7	5.59 (m)	123.76	C-6, 9	H-6, 8
8	5.59 (m)	134.52	C-6, 9	H-9
9	2.19 (m)	32.15	ccC-7, 8, 10, 11	H-8, 10
10	1.64 (m)	27.45	C-8, 9, 11, 12	H-9, 11
11	2.28 (td, 6.99, 1.29)	18.84	C-9, 10, 12, 13, 14, 15	H-10
12	-	77.85	-	-
13	-	68.48	-	-
14	-	68.59	-	-
15	1.97 (t, 1.18)	65.16	-	-

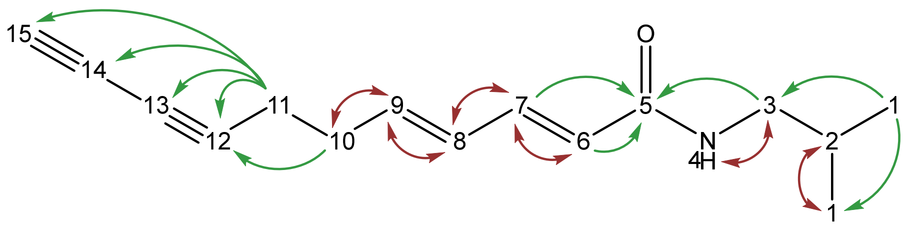

^{1}H NMR ($CDCl_3$ with 0.03% v/v TMS, 700 MHz): d_H 0.93 (d, 6.67, H1), 1.81 (m, 13.44, 6.67, H2), 3.14 (dd, 6.88, 613, H3), 5.50 (bs, H4), 5.52 (d, 11.29, H6), 6.38 (t, 11.24, H7), 7.53 (m, H8), 5.97 (dddd, 14.29, 5.16, 4.04, 1.11, H9), 2.42 (m, H10), 2.39 (m, H11), 1.98 (m, H15). ^{13}C NMR ($CDCl_3$, 175 MHz): d_C 166.41, 119.72, 140.97, 128.10, 139.72, 31.30, 19.16, 77.57, 68.47, 68.47, 65.50.

Figure S7A. Compound 2. *N*-isobutylundeca-2(*E*),4(*E*)-diene-8,10-dynamide.

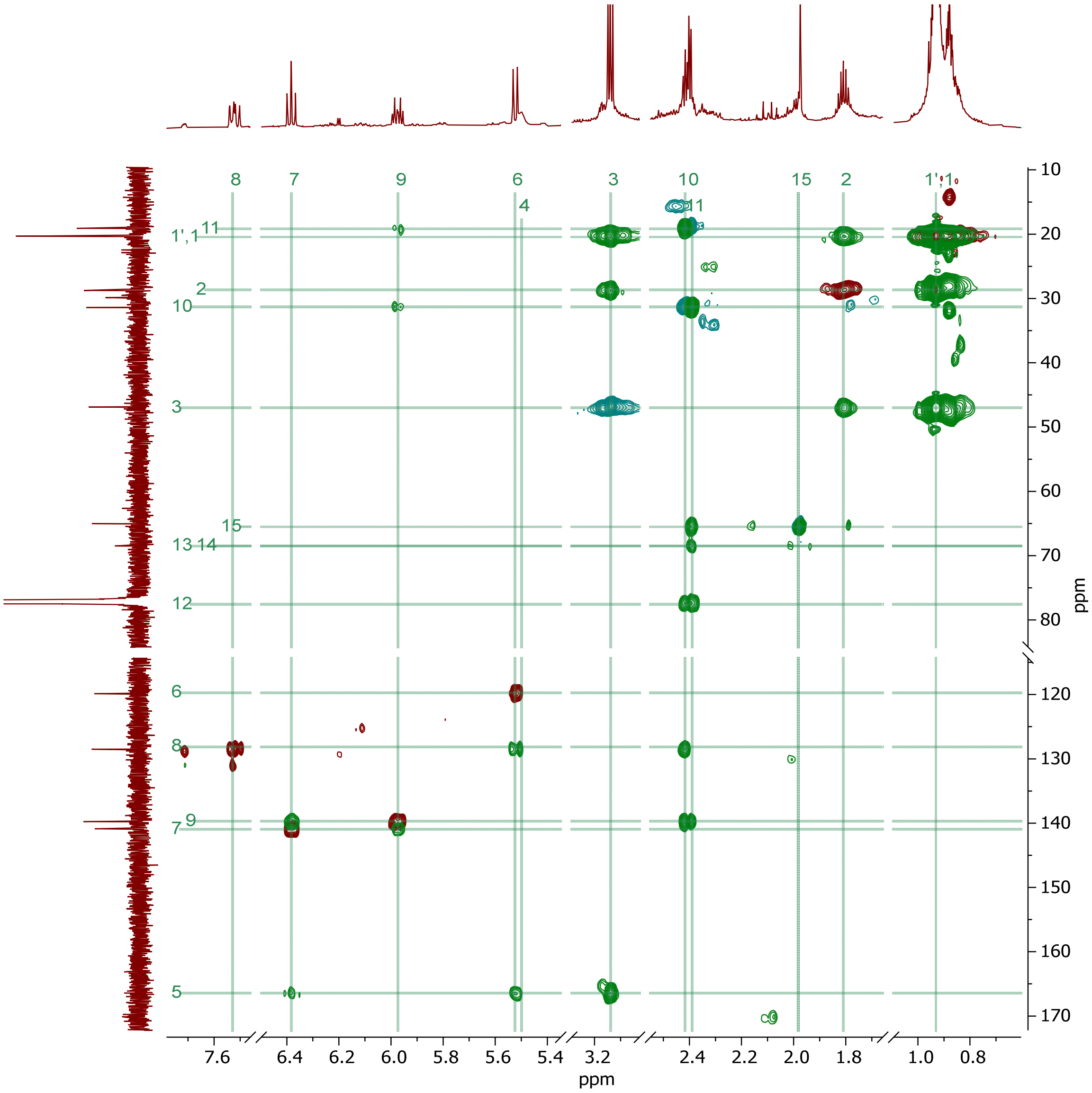

Figure S7B. ed-HSQC (red - blue) and HMBC (green) spectra (700 MHz, 300 K, $CDCl_3$).

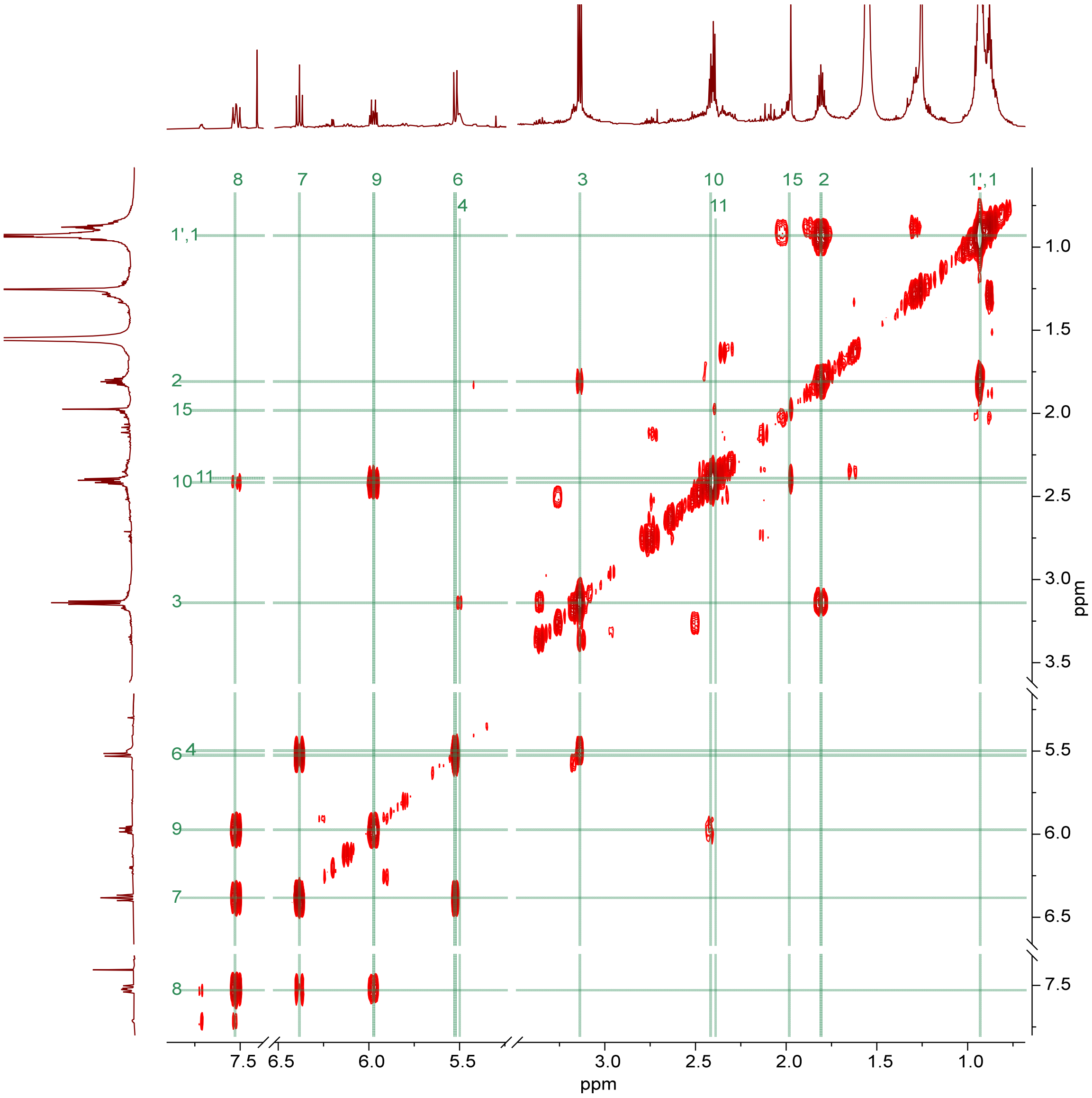

Figure S7C. COSY spectra (700 MHz, 300 K, $CDCl_3$).

Table S7. Spectroscopic data: d_H, d_C, HMBC and COSY (300 K, $CDCl_3$).

Assignation	δH/ppm (Multiplicity, J/Hz)	δC/ppm	HMBC (H→C)	COSY (H→H)
1	0.93 (d, 6.67)	20.40	C-1, 2, 3	H-2
2	1.81 (m, 13.44, 6.67)	28.68	C-1, 3	H-1, 3
3	3.14 (dd, 6.88, 613)	46.99	C-1, 2, 5	H-2, 4
4	5.50 (bs)	-	-	H-3
5	-	166.41	-	-

6	5.52 (d, 11.29)	119.72	C-5, 8	H-7
7	6.38 (t, 11.24)	140.97	C-5, 9	H-6, 8
8	7.53 (m)	128.10	-	H-7, 9
9	5.97 (dddd, 14.29, 5.16, 4.04, 1.11)	139.72	C-10, 11	H-8, 10
10	2.42 (m)	31.30	C-8, 9, 11, 12	H-9
11	2.39 (m)	19.16	C-9, 10, 13, 14, 15	-
12	-	77.57	-	-
13	-	68.47	-	-
14	-	68.47	-	-
15	1.98 (m)	65.50	-	-

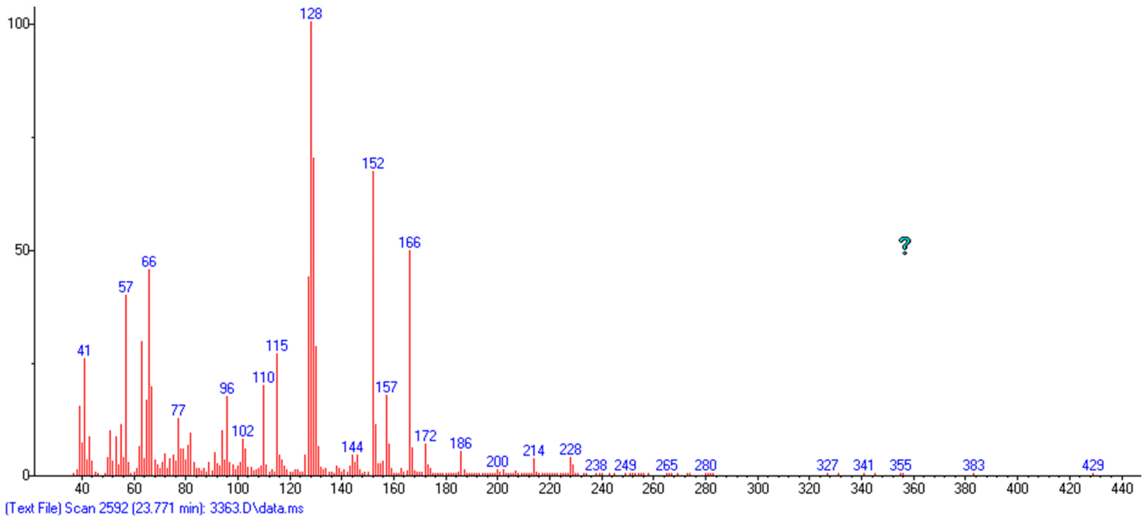

Figure S7D. Mass spectrum (EI, 70 eV) of compound 2 (*m/z* 33–600).

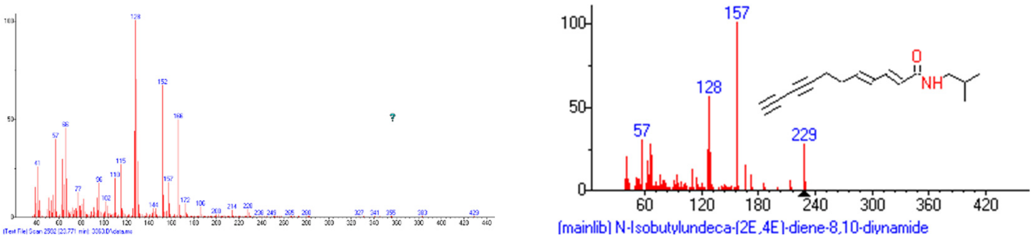

Figure S7E. Comparison of the experimental mass spectrum of compound 2 (A) with the NIST23 library reference for *N*-isobutylundeca-2(E),4(Z)-diene-8,10-dynamide (B).

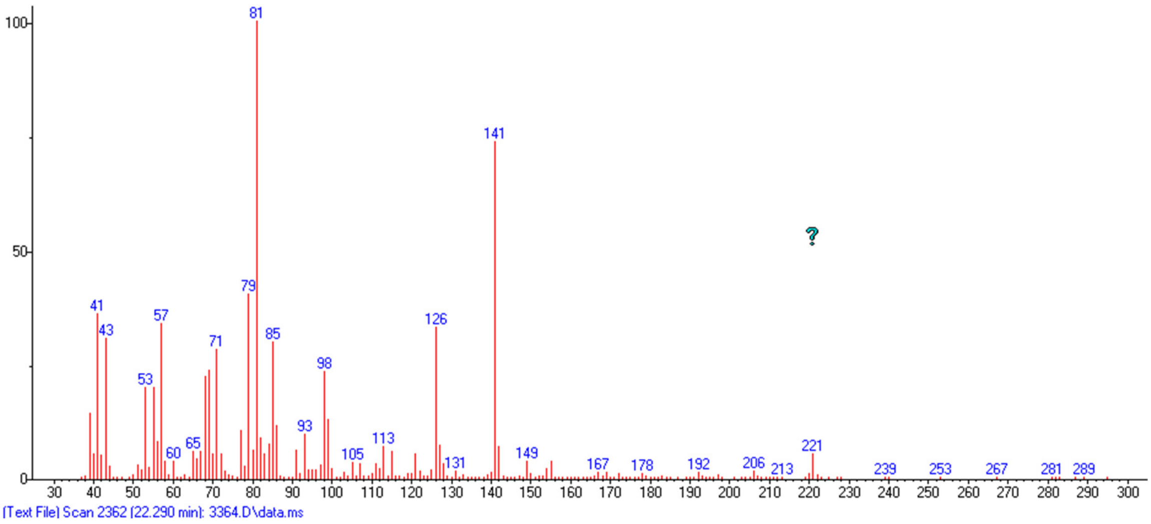

Figure S8A. Mass spectrum (EI, 70 eV) of compound 3 (m/z 33–600).

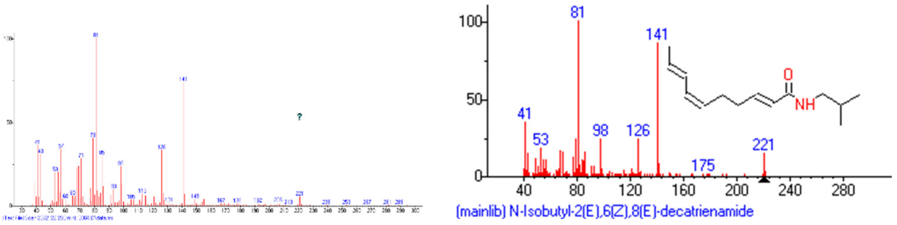

Figure S8B. Comparison of the experimental mass spectrum of compound 3 (A) with the NIST23 library reference for *N*-isobutyl-2(*E*),6(*Z*),8(*E*)-decatrienamide (B).

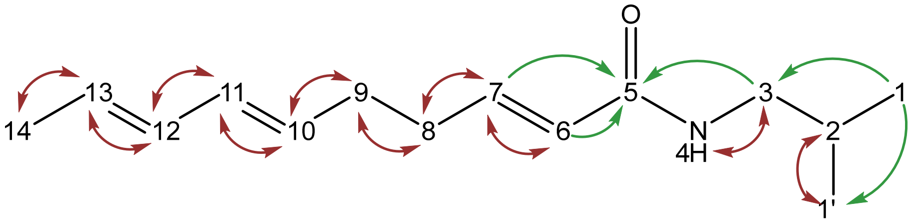

^{1}H NMR ($CDCl_3$ with 0.03% v/v TMS, 700 MHz): d_H 0.92 (d, 6.70, H1), 1.79 (m, H2), 3.14 (dd, 6.90, 6.09, H3), 5.51 (bs,H4), 5.79 (dt, 15.27, 1.59, H6), 6.81 (dt, 15.25, 6.71, H7) 2.25 (m, H8), 2.31 (q, 7.27, 6.88, H9), 5.25 (dtd 10.86, 7.42, 7.37, 0.91, H10), 5.96 (10.95, H11), 6.28 (dddt, 15.49, 10.96, 3.19, 1.67, H12), 5.69 (dq, 13.81, 6.75, H13) 1.77 (m, H14). ^{13}C NMR ($CDCl_3$, 175 MHz): d_C 20.36, 28.50, 46.96, 165.89, 124.25, 143.72, 32.12, 26.60, 127.80, 129.53, 126.80, 130.13, 18.43.

Figure S9A. Compound 4: *N*-isobutyl-2(*E*),6(*Z*),8(*E*)-decatrienamide

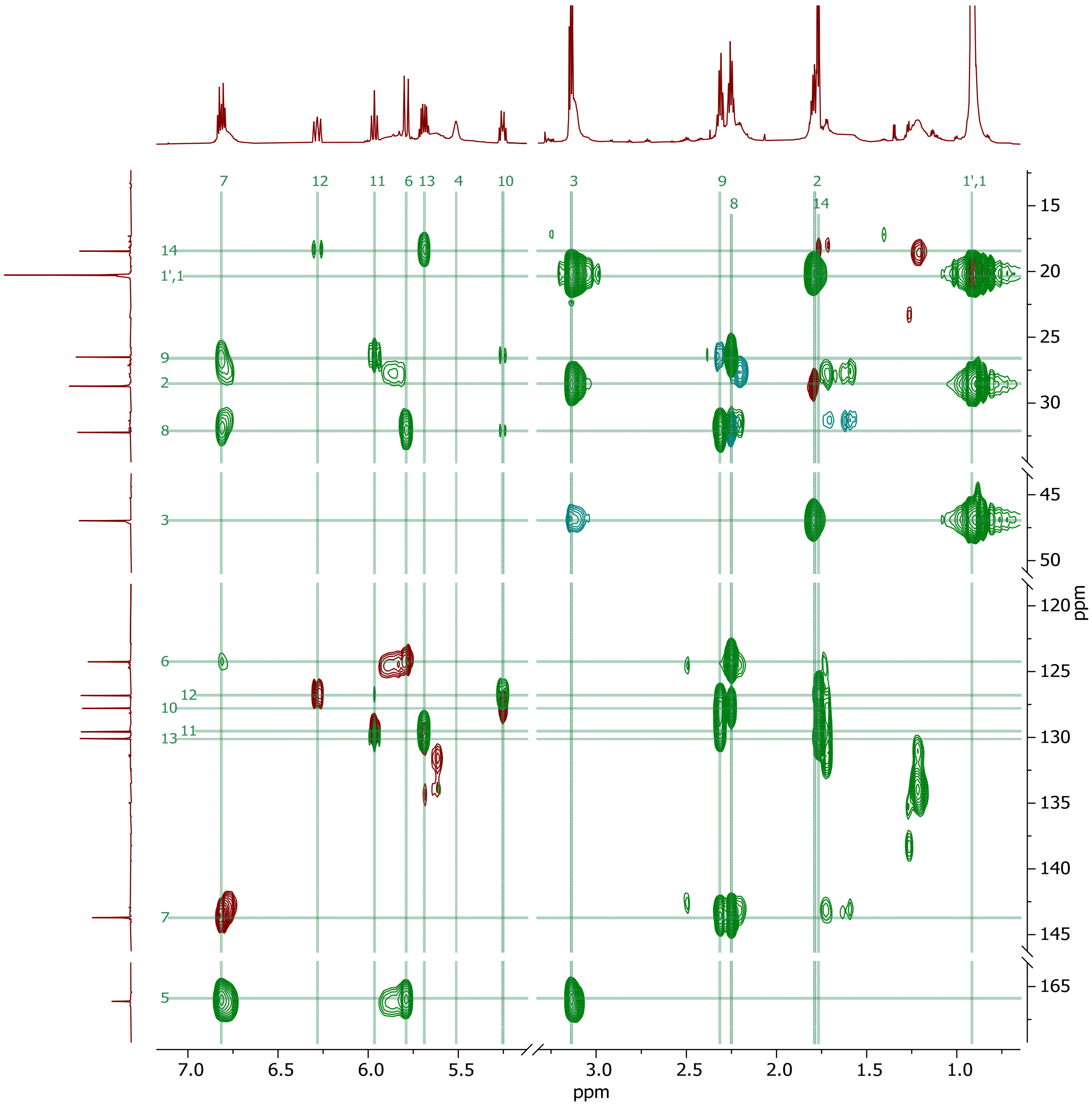

Figure S9B. ed-HSQC (red - blue) and HMBC (green) spectra (700 MHz, 300 K, $CDCl_3$).

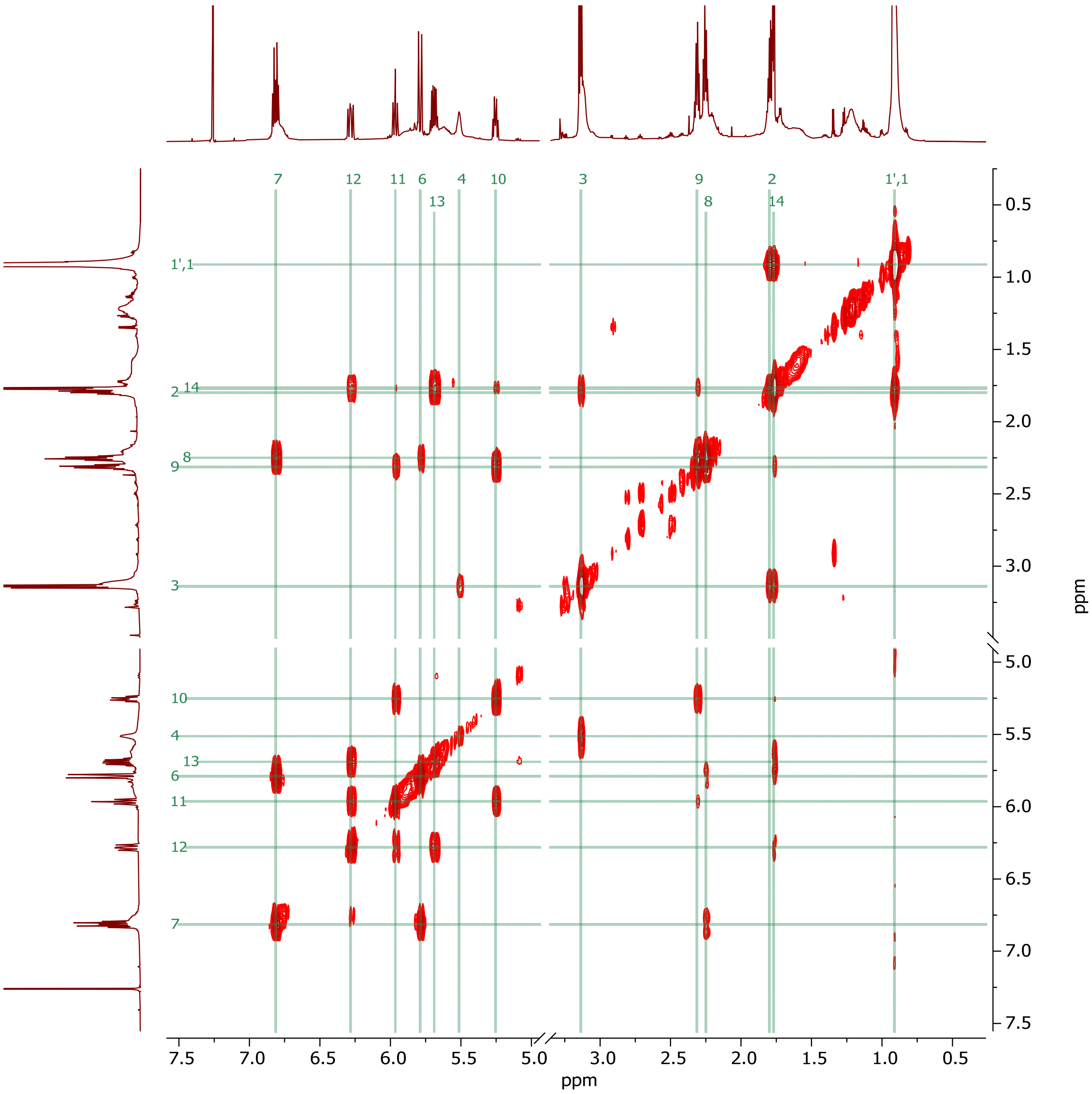

Figure S9C. COSY spectra (700 MHz, 300 K, $CDCl_3$).

Table S8. Spectroscopic data: d_H, d_C, HMBC and COSY (300 K, $CDCl_3$).

Assignation	δH/ppm (Multiplicity, J/Hz)	δC/ppm	HMBC (H→C)	COSY (H→H)
1	0.92 (d, 6.70)	20.36	C-1, 2, 3	H-2
2	1.79 (m)	28.50	C-1, 3	H-1, 3
3	3.14 (dd, 6.90, 6.09)	46.96	C-1, 2, 5	H-2, 4
4	5.51 (bs)	-	-	H-3
5	-	165.89	-	-

6	5.79 (dt, 15.27, 1.59)	124.25	C-5, 8	H-7
7	6.81 (dt, 15.25, 6.71)	143.72	C-5, 8, 9	H-6, 8
8	2.25 (m)	32.12	C-6, 7, 9, 10	H-7, 9
9	2.31 (q, 7.27, 6.88)	26.60	C-7, 8, 10, 11	H-8, 10
10	5.25 (dtd 10.86, 7.42, 7.37, 0.91)	127.80	C-8, 9, 11, 12	H-9, 11
11	5.96 (10.95)	129.53	C-9, 12, 13	H-10, 12
12	6.28 (dddt, 15.49, 10.96, 3.19, 1.67)	126.80	C-10,11, 13, 14	H-11, 13
13	5.69 (dq, 13.81, 6.75)	130.13	C-11, 14	H-12, 14
14	1.77 (m)	18.43	C-12, 13	H-13

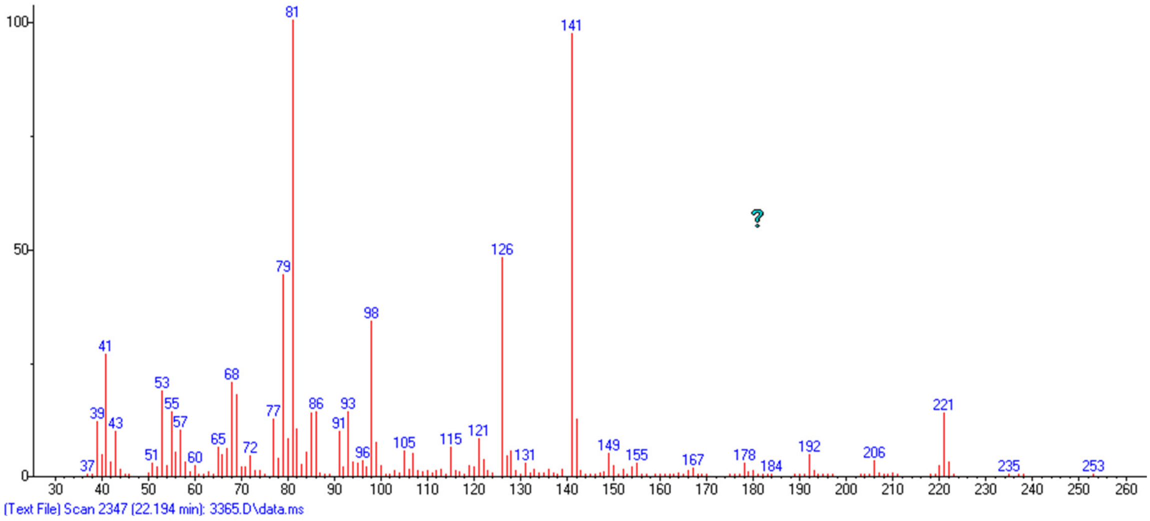

Figure S9D. Mass spectrum (EI, 70 eV) of compound 4 (*m/z* 33–600).

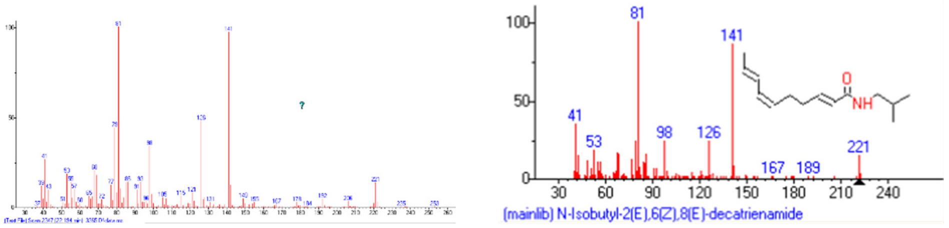

Figure S9E. Comparison of the experimental mass spectrum of compound 4 (A) with the NIST23 library reference for *N*-isobutyl-2(*E*),6(*Z*),8(*E*)-decatrienamide (B).

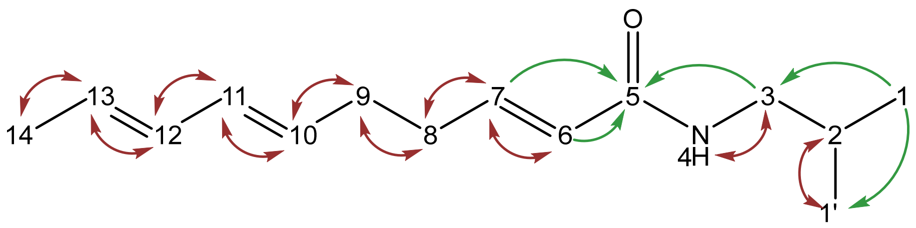

^{1}H NMR ($CDCl_3$ with 0.03% v/v TMS, 700 MHz): d_H 0.94 (d, 6.72, H1) 1.82 (m, H2) 3.17 (t, 6.48, H3) 5.44 (bs, H4), 5.80 (m, H6), 6.84 (m, H7) 2.29 (m, H8) 2.34 (m, H9) 5.26 (m, H10) 5.98 (m, H11) 6.29 (m, H12) 5.70 (m, H13) 1.76 (d, 6.98, H14). ^{13}C NMR ($CDCl_3$, 175 MHz): d_C 20.20, 28.60, 46.94, 165.89, 123.88, 143.38, 31.67, 27.74, 127.67, 129.71, 126.66, 130.75, 17.89.

Figure S10A. Compound 5: *N*-isobutyl-2(*E*),6(*Z*),8(*E*)-decatrienamide

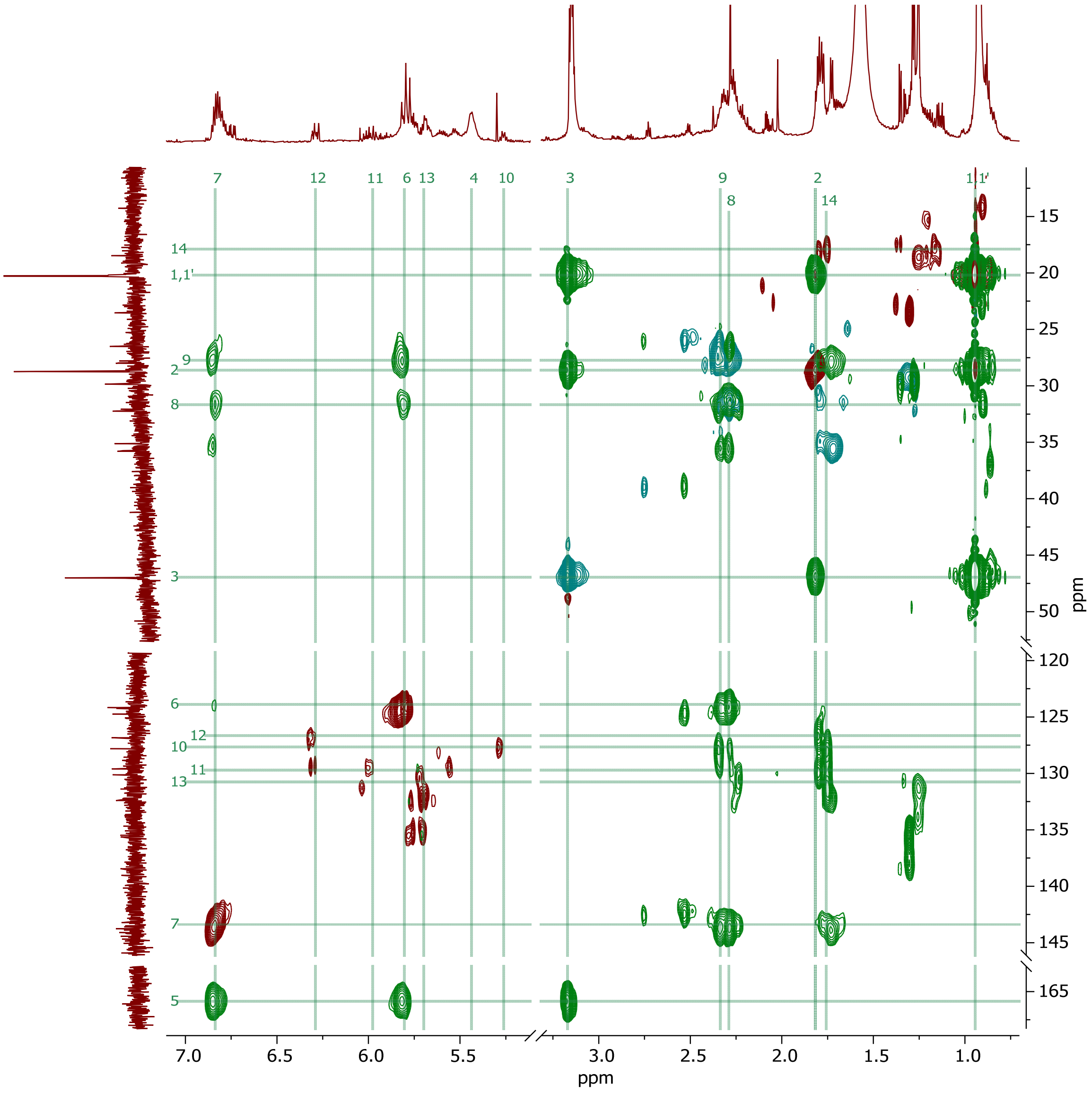

Figure S10B. ed-HSQC (red - blue) and HMBC (green) spectra (700 MHz, 300 K, $CDCl_3$).

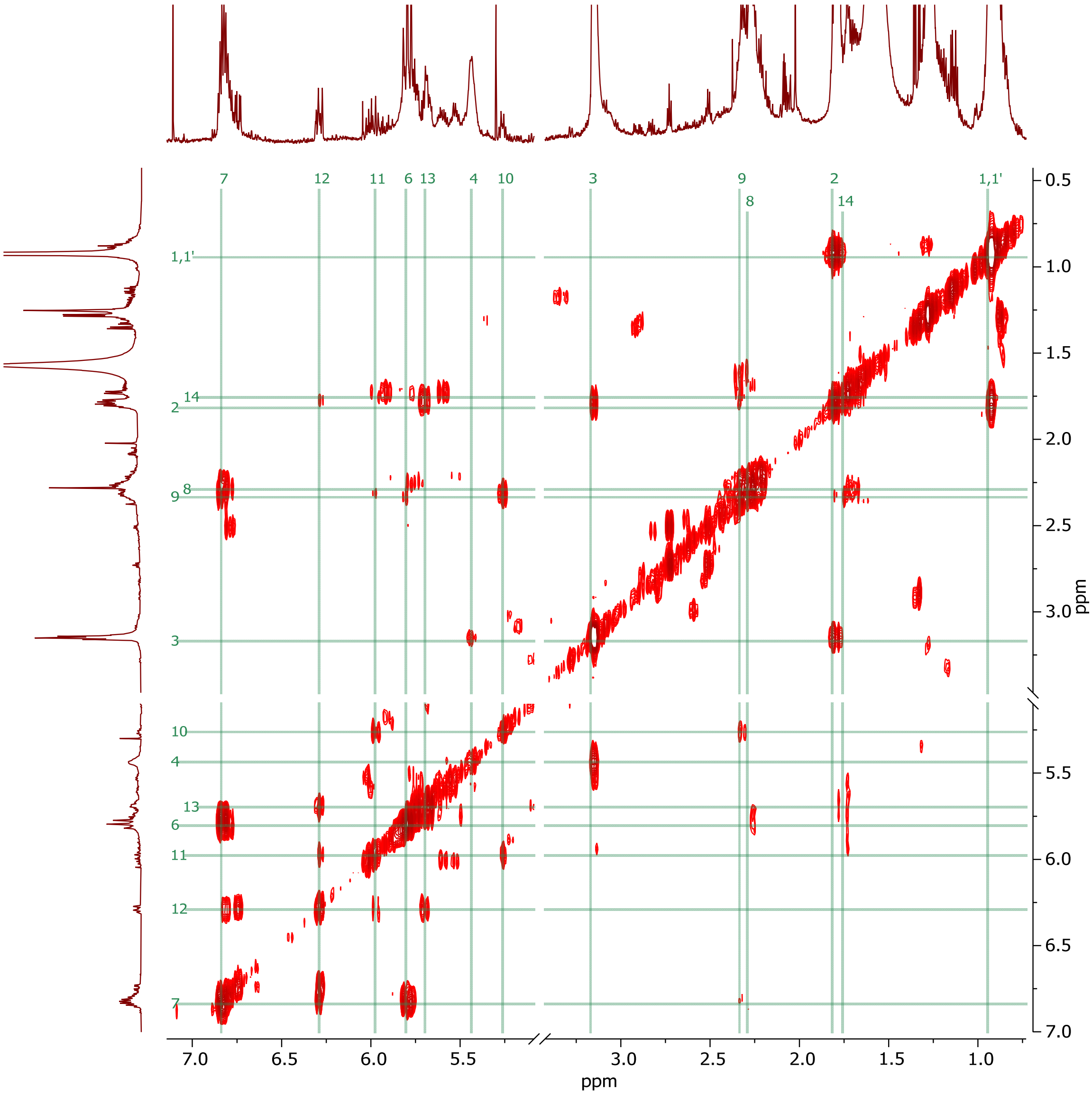

Figure S10C. COSY spectra (700 MHz, 300 K, $CDCl_3$).

Table S9. Spectroscopic data: d_H, d_C, HMBC and COSY (300 K, $CDCl_3$).

Assignation	δH/ppm (Multiplicity, J/Hz)	δC/ppm	HMBC (H→C)	COSY (H→H)
1	0.94 (d, 6.72)	20.20	C-1, 2, 3	H-2
2	1.82 (m)	28.60	C-1, 3	H-1, 3
3	3.17 (t, 6.48)	46.94	C-1, 2, 5	H-2, 4
4	5.44 (bs)	-	-	H-3

5	-	165.89	-	-
6	5.80 (m)	123.88	C-5, 8	H-7
7	6.84 (m)	143.38	C-5, 8, 9	H-6, 8
8	2.29 (m)	31.67	C-6, 7, 9, 10	H-7, 9
9	2.34 (m)	27.74	C-7, 8, 10, 11	H-8, 10
10	5.26 (m)	127.67	-	H-9, 11
11	5.98 (m)	129.71	-	H-10, 12
12	6.29 (m)	126.66	-	H-11, 13
13	5.70 (m)	130.75	C-14	H-12, 14
14	1.76 (d, 6.98)	17.89	C-12, 13	H-13

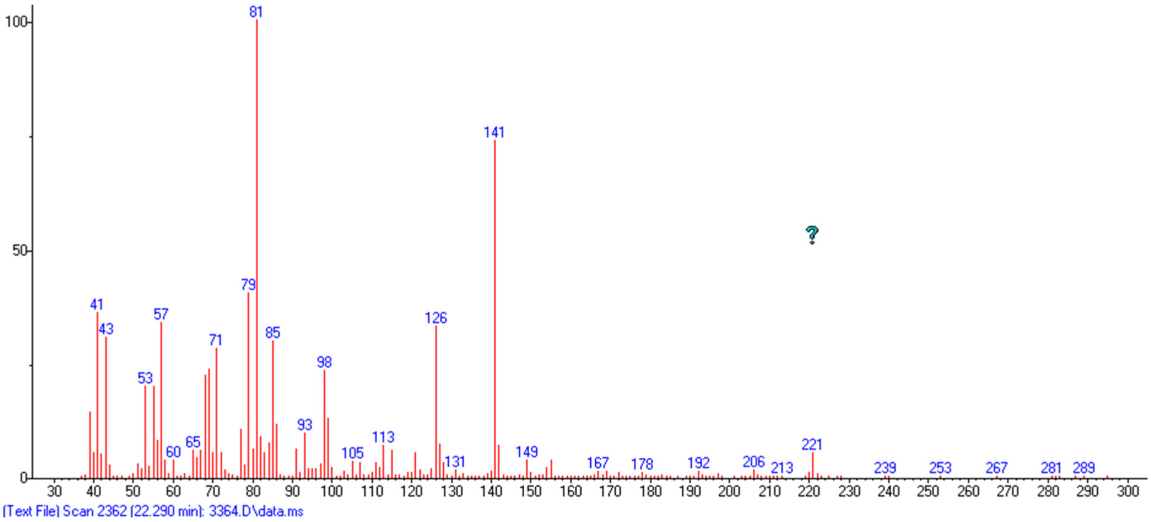

Figure S10D. Mass spectrum (EI, 70 eV) of compound 4 (*m/z* 33–600).

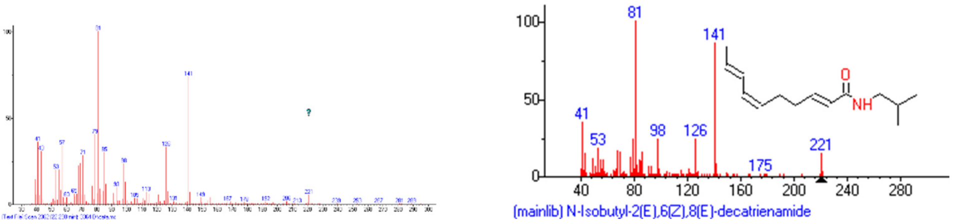

Figure S10E. Comparison of the experimental mass spectrum of compound 5 (A) with the NIST23 library reference for *N*-isobutyl-2(*E*),6(*Z*),8(*E*)-decatrienamide (B).

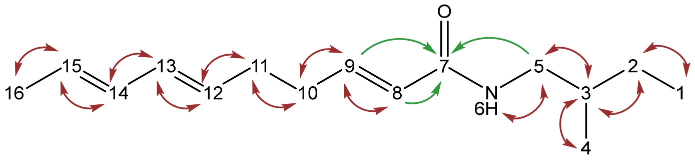

^{1}H NMR ($CDCl_3$ with 0.03% v/v TMS, 700 MHz): d$_H$ 0.89 (m, H1), 1.14, 1.41 (m, H2), 1.58 (m, H3), 0.89 (m, H4), 3.13, 3.25 (m, H5), 5.48 (m, H6), 5.79 (dt, 15.28, 1.60, H8), 6.81 (m, H9), 2.25 (m, H10), 2.32 (m, H11), 5.25 (dt, 10.96, 7.26, H12), 5.98 (t, 11.03, H13), 6.29 (ddp, 15.54, 10.90, 1.68,1.65, H14), 5.69 (qd, 13.80, 6.71, H15), 1.77 (dd, 6.79, 1.64, H16). ^{13}C NMR ($CDCl_3$, 175 MHz): d$_C$ 11.11, 27.37, 35.17, 17.02, 45.00, 166.20, 124.19, 143.21, 32.08, 26.51, 127.86, 129.49, 126.85, 130.27, 18.52.

Figure S11A. Compound 6: *N*-(2-Methylbutyl)-2(*E*),6(*Z*),8(*E*)-decatrienamide

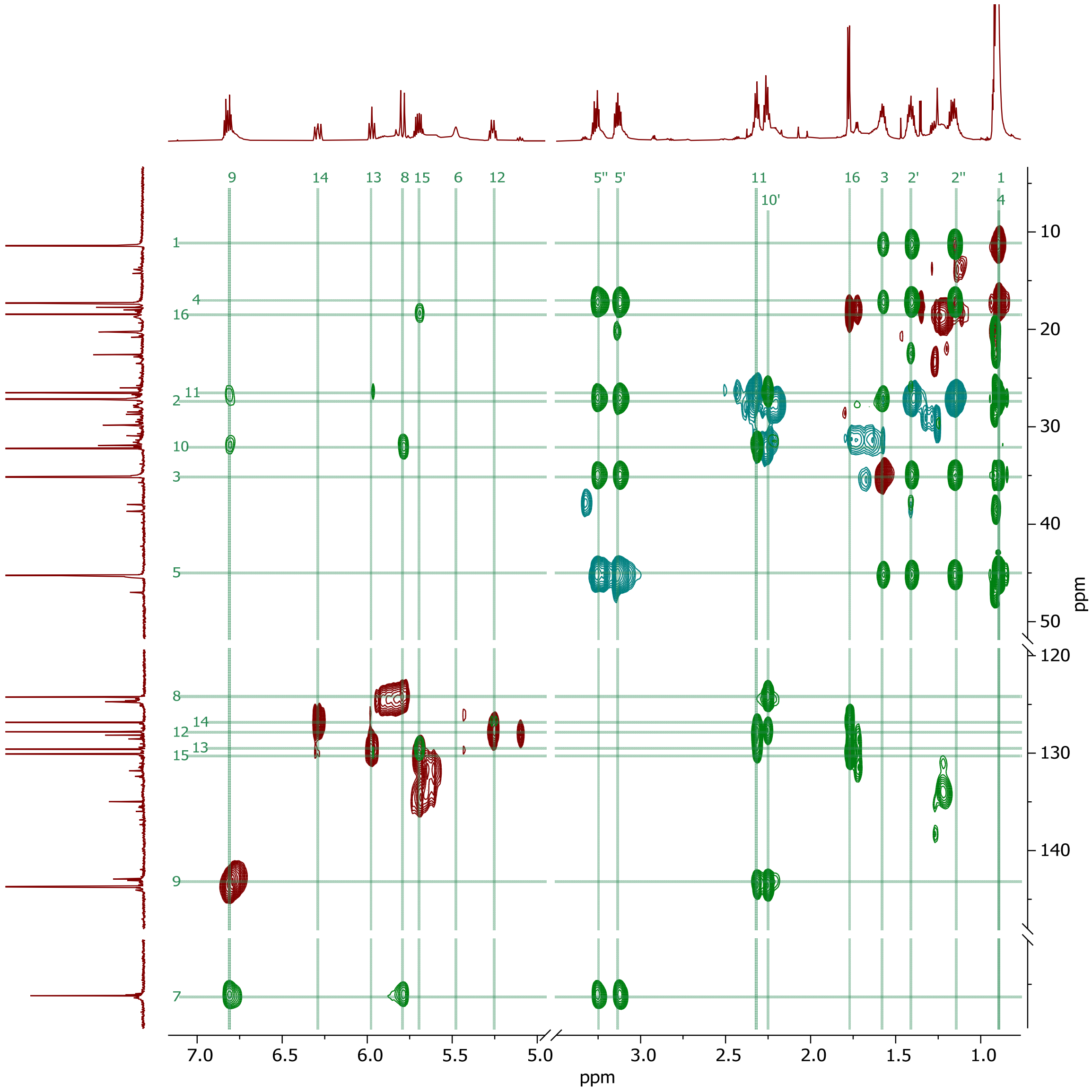

Figure S11B. ed-HSQC (red - blue) and HMBC (green) spectra (700 MHz, 300 K, $CDCl_3$).

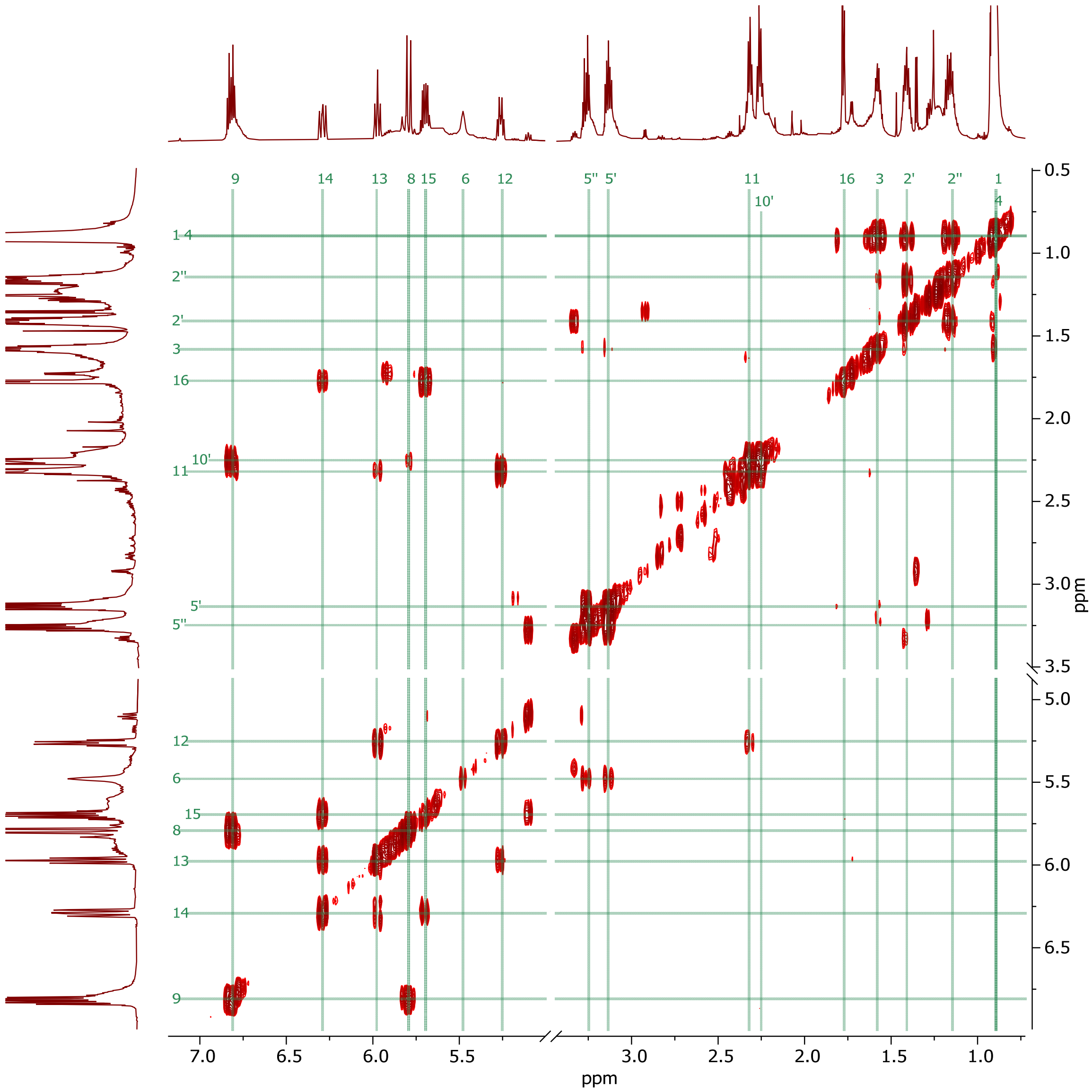

Figure S11C. COSY spectra (700 MHz, 300 K, $CDCl_3$).

Table S10. Spectroscopic data: d_H, d_C, HMBC and COSY (300 K, $CDCl_3$).

Assignation	δH/ppm (Multiplicity, J/Hz)	δC/ppm	HMBC (H→C)	COSY (H→H)
1	0.89 (m)	11.11	C-2, 3	H-2
2, 2’	1.14, 1.41 (m)	27.37	C-1, 3, 4, 5	H-1, 3
3	1.58 (m)	35.17	C-1, 2, 4, 5	H-2, 4, 5
4	0.89 (m)	17.02	C-2, 3, 5	H-3
5, 5’	3.13, 3.25 (m)	45.00	C-2, 3, 4, 7	H-3-

6	5.48 (m)	-	-	-
7	-	166.20	-	-
8	5.79 (dt, 15.28, 1.60)	124.19	C-7, 10	H-9
9	6.81 (m)	143.21	C-7, 10, 11	H-8, 10
10	2.25 (m)	32.08	C-8, 9, 11, 12	H-9, 11
11	2.32 (m)	26.51	C-9, 10, 11, 12	H-10, 12
12	5.25 (dt, 10.96, 7.26)	127.86	C-10, 11, 14	H-11, 13
13	5.98 (t, 11.03)	129.49	C-11, 14, 15	H-12, 14
14	6.29 (ddp, 15.54, 10.90, 1.68,1.65)	126.85	C-13, 16	H-13, 15
15	5.69 (qd, 13.80, 6.71)	130.27	C-13, 16	H-14, 16
16	1.77 (dd, 6.79, 1.64)	18.52	C-14, 15	H-15

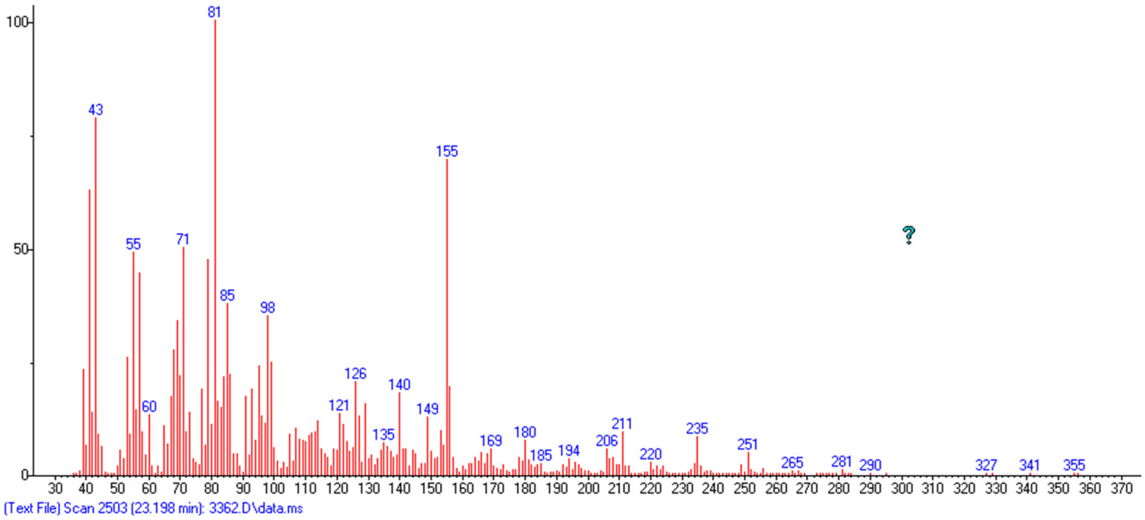

Figure S11D. Mass spectrum (EI, 70 eV) of compound 6 (*m/z* 33–600).

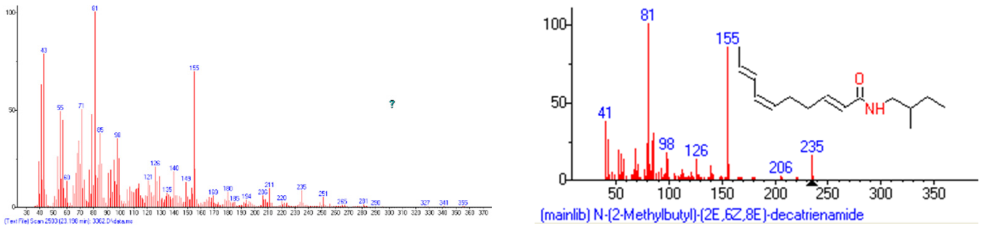

Figure S11E. Comparison of the experimental mass spectrum of compound 5 (A) with the NIST23 library reference for *N*-(2-Methylbutyl)-2(*E*),6(*Z*),8(*E*)-decatrienamide (B).

Table S11A. Chemical profile of non-polar compounds identified by GC-MS in the vegetative stage of *Heliopsis longipes*.

Name	Biosynthetic family	Match	% Area	CAS number
N-Isobutyl-2(*E*),6(*Z*),8(*E*)-decatrienamide	Alkamide	70	10.490	1000106-17-5
N-Isobutylundeca-2(*E*)-en-8,10-diynamide	Alkamide	50	3.1102	099615-81-3
13-Docosenamide, (*Z*)-	Alkamide	99	1.3625	000112-84-5
Crotonic acid	Fatty acid-derived compounds	96	0.4009	000541-47-9
n-Capric acid	Fatty acid	95	0.0675	000334-48-5
Palmitic acid	Fatty acid	99	0.9880	000057-10-3
Methyl stearate	Fatty acid-derived compounds	96	1.1698	000112-61-8
Octan-2-yl palmitate	Fatty acid-derived compounds	90	1.0839	055194-81-5
2-Pentadecanone, 6,10,14-trimethyl	Isoprenoid	98	0.2164	000502-69-2
Benzaldehyde	Phenylpropanoid	87	0.0228	000100-52-7
Coumarin, 8-methoxy-	Phenylpropanoid	87	0.1921	002445-81-0
.beta.-sitosterol	Phytosterol	95	0.0102	000083-46-5
(+)-sesamin	Lignane	99	2.5974	000607-80-7
Fargesin	Lignane	99	1.3575	031008-19-2
n-eicosane	Lipid-derived compounds	90	0.0006	000112-95-8
Hexadecane	Lipid-derived compounds	91	1.2417	000544-76-3
n-pentadecane	Lipid-derived compounds	95	0.3265	000629-62-9
Tricosane	Lipid-derived compounds	94	1.0306	000638-67-5
Pentadecane, 2-methyl-	Lipid-derived compounds	91	3.3776	001560-93-6
Octadecane	Lipid-derived compounds	93	1.0801	000593-45-3
1-docosene	Lipid-derived compounds	95	3.0186	001599-67-3
Hexacosane	Lipid-derived compounds	94	1.7077	000630-01-3
Nonadecane	Lipid-derived compounds	91	1.0961	000629-92-5
Octacosane	Lipid-derived compounds	99	3.8291	000630-02-4
Docosane	Lipid-derived compounds	94	1.5160	000629-97-0
Heptacosane	Lipid-derived compounds	96	4.5665	000593-49-7
n-pentacosane	Lipid-derived compounds	93	2.5159	000629-99-2
2-methylpentacosane	Lipid-derived compounds	93	2.4653	000629-87-8
Tetracosane	Lipid-derived compounds	98	3.447	000646-31-1
Hentriacontane	Lipid-derived compounds	90	1.1578	000630-04-6
Heptadecane, 2-methyl-	Lipid-derived compounds	94	0.8796	001560-89-0
10-methyldotriacontane	Lipid-derived compounds	90	1.0703	058349-83-0
Heneicosane	Lipid-derived compounds	97	1.4611	000629-94-7

Triacontane	Lipid-derived compounds	89	0.5959	000638-68-6
Squalene	Terpene	83	1.2354	000111-02-4
Lup-20(29)-en-3-one	Terpene	99	0.1912	001617-70-5

Note: Identification based on comparison of mass spectra and CAS numbers with the NIST® 2023 library.

Table S11B. Chemical profile of non-polar compounds identified by GC-MS in the flowering stage of *Heliopsis longipes*.

Name	Biosynthetic family	Match	% Area	CAS number
N-Isobutyl-2(*E*),6(*Z*),8(*E*)-decatrienamide	Alkamide	81	18.2281	1000106-17-5
N-(2-Methylbutyl)-(2*E*,6*Z*,8*E*)-decatrienamide	Alkamide	93	2.8627	1010106-16-0
Hexadecanamide	Alkamide	89	1.3850	000629-54-9
Benzaldehyde	Phenylpropanoid	87	0.077	000100-52-7
Stigmastan-3,5,22-trien	Phytosterol	89	0.1306	100214-18-1
Stigmasta-3,5-diene	Phytosterol	98	0.2720	004970-37-0
Stigmasterol	Phytosterol	86	0.0827	000083-48-7
(+)-sesamin	Lignane	99	1.4199	000607-80-7
Farnesane	Lipid-derived compounds	86	0.2442	014905-56-7
Pentadecane	Lipid-derived compounds	91	1.1906	000629-62-9
Heptadecane	Lipid-derived compounds	93	0.2474	000629-78-7
Pristane	Lipid-derived compounds	90	0.1471	000638-36-8
Octadecane	Lipid-derived compounds	95	1.0056	000593-45-3
Nonadecane	Lipid-derived compounds	91	1.6541	000629-92-5
Heneicosane	Lipid-derived compounds	94	4.1944	000629-94-7
Docosane	Lipid-derived compounds	91	4.9778	000629-97-0
Heptacosane	Lipid-derived compounds	97	2.5118	000593-49-7
2-methylhentriacontane	Lipid-derived compounds	95	4.9779	000629-97-0
Hexacosane	Lipid-derived compounds	99	2.5119	000593-49-7
n-pentacosane	Lipid-derived compounds	96	0.9993	001720-12-3
Octacosane	Lipid-derived compounds	99	1.4586	000630-01-3
Hexadecane	Lipid-derived compounds	94	1.0210	000629-99-2
n-eicosane	Lipid-derived compounds	94	1.0329	000630-02-4
Tetracosane	Lipid-derived compounds	98	0.1598	000112-95-8
Lup-20(29)-en-3-one	Terpene	74	0.0356	001617-70-5

Note: Identification based on comparison of mass spectra and CAS numbers with the NIST® 2023 library.

Table S11C. Chemical profile of non-polar compounds identified by GC-MS in the fruit setting stage of *Heliopsis longipes*.

Name	Biosynthetic family	Match	% Area	CAS number
N-Isobutyl-2(*E*),6(*Z*),8(*E*)-decatrienamide	Alkamide	76	19.4572	1000106-17-5
N-(2-Methylbutyl)-(2*E*,6*Z*,8*E*)-decatrienamide	Alkamide	81	3.2131	1010106-16-0
N-isobutylhexadecanamide	Alkamide	90	8.7655	054794-72-8
Benzaldehyde	Phenylpropanoid	96	0.077	000100-52-7
Stigmasta-3,5-diene	Phytosterol	99	0.5658	004970-37-0
Stigmasterol	Phytosterol	99	0.5176	000083-48-7
.gamma.-sitosterol	Phytosterol	93	1.0354	000083-47-6
(+)-sesamin	Lignane	97	1.9082	000607-80-7
Dodecane	Lipid-derived compounds	90	0.8925	000112-40-3
Pentadecane	Lipid-derived compounds	87	0.2930	000629-62-9
Hexadecane	Lipid-derived compounds	93	0.1043	000544-76-3
Heptadecane	Lipid-derived compounds	94	0.2277	000629-78-7
Heptacosane	Lipid-derived compounds	90	1.3969	000593-49-7
Octadecane	Lipid-derived compounds	93	0.6537	000593-45-3
n-eicosane	Lipid-derived compounds	94	4.2717	000112-95-8
Heneicosane	Lipid-derived compounds	97	2.1913	000629-94-7
Triacontane	Lipid-derived compounds	96	1.0606	000638-68-6
Tetracosane	Lipid-derived compounds	83	0.0966	000646-31-1
Erucamide	Lipid-derived compounds	86	0.3619	000112-84-5
Docosane	Lipid-derived compounds	90	0.1618	000629-97-0
n-pentacosane	Lipid-derived compounds	93	0.0148	000629-99-2
Hentriacontane	Lipid-derived compounds	81	0.0887	000630-04-6
Octacosane	Lipid-derived compounds	89	0.0796	000630-02-4
3-Ethyl-2,6,10-trimethylundecane	Lipid-derived compounds	80	0.063	1000432-25-9
Squalene	Terpene	86	0.1037	007683-64-9
.alpha.-tocospiro B	Terpene	91	0.02818	601490-41-9
dl-.alpha.-tocopherol	Terpene	89	0.0009	010191-41-0
Lup-20(29)-en-3-one	Terpene	99	0.2249	001617-70-5

Note: Identification based on comparison of mass spectra and CAS numbers with the NIST® 2023 library.

Table S11D. Chemical profile of non-polar compounds identified by GC-MS in the defoliation stage of *Heliopsis longipes*.

Name	Biosynthetic family	Match	% Area	CAS number
N-Isobutyl-2(*E*),6(*Z*),8(*E*)-decatrienamide	Alkamide	87	34.1552	1000106-17-5

N-(2-Methylbutyl)-(2*E*,6*Z*,8*E*)-decatrienamide	Alkamide	90	6.0113	1010106-16-0
N-isobutylhexadecanamide	Alkamide	49	1.1899	054794-72-8
Cetyl palmitate	Fatty acid-derived compounds	86	0.0717	000540-10-3
(+)-sesamin	Lignane	91	0.3214	000133-03-9
1-dodecene	Lipid-derived compounds	96	0.1627	000112-41-4
Pentadecane	Lipid-derived compounds	87	0.8052	000629-62-9
Hexadecane	Lipid-derived compounds	93	0.3176	000544-76-3
Dodecane, 2-methyl-8-propyl-	Lipid-derived compounds	86	0.2868	055045-07-3
Heptadecane, 2-methyl-	Lipid-derived compounds	91	0.0629	001560-89-0
Heptadecane	Lipid-derived compounds	97	0.4994	000629-78-7
Pristano	Lipid-derived compounds	91	0.2084	001921-70-6
Docosane	Lipid-derived compounds	87	1.8738	000629-97-0
4-methoxy-7-methylindan-1-one	Lipid-derived compounds	89	0.1858	103988-25-6
Heptacosane	Lipid-derived compounds	90	0.6693	000593-49-7
n-eicosane	Lipid-derived compounds	91	0.2277	000112-95-8
Heneicosane	Lipid-derived compounds	80	0.3967	000629-94-7
2-methylhexacosane	Lipid-derived compounds	83	0.3413	001561-02-0
Hexadecane, 2,6,10,14-tetramethyl-	Lipid-derived compounds	93	1.0618	000638-36-8
Isopropyl myristate	Lipid-derived compounds	96	0.6484	000110-27-0
Triacontane	Lipid-derived compounds	86	1.1699	000638-68-6
trans-2,3-Epoxydecane	Lipid-derived compounds	83	1.4186	054125-39-2
11-tricosene	Lipid-derived compounds	93	4.2798	052078-56-5
Octadecane	Lipid-derived compounds	95	1.8486	000593-45-3
Nonacos-1-ene	Lipid-derived compounds	95	6.1775	018835-35-3
Docosane	Lipid-derived compounds	94	0.5541	000629-97-0
11-methylpentacosane	Lipid-derived compounds	83	0.6545	015689-71-1
n-pentacosane	Lipid-derived compounds	98	1.2066	000629-99-2
Hexacosane	Lipid-derived compounds	99	1.2054	000630-01-3
Heptacosane	Lipid-derived compounds	80	0.1447	000593-49-7
Tetracosane	Lipid-derived compounds	97	0.9066	000646-31-1
9-octadecenamide, (Z)-	Lipid-derived compounds	89	0.4028	000301-02-0
Hexadecane, 3-methyl-	Lipid-derived compounds	91	0.1054	006418-43-5
Hexadecane, 2-methyl-	Lipid-derived compounds	90	0.1022	001560-92-5
Heptadecane, 3-methyl-	Lipid-derived compounds	93	0.0740	006418-44-6
Heptatriacontane	Lipid-derived compounds	91	0.0331	007194-84-5
Heptadecane, 9-octyl-	Lipid-derived compounds	81	0.1007	007225-64-1
Benzaldehyde	Phenylpropanoid	96	0.077	000100-52-7
Stigmasta-3,5-diene	Phytosterol	91	0.3921	004970-37-0
Stigmasterol	Phytosterol	98	0.7819	000083-48-7
.gamma.-Sitosterol	Phytosterol	95	0.2581	000083-47-6
Cycloartenol acetate	Terpene	80	0.0401	001259-10-5
D-limonene	Terpene	93	0.2338	005989-27-5
Squalene	Terpene	97	0.0582	007683-64-9
.alpha.-tocospiro A	Terpene	95	0.2811	601490-40-8
dl-.alpha.-tocopherol	Terpene	89	0.0604	000059-02-9
Lup-20(29)-en-3-one	Terpene	85	0.0907	001617-70-5

Note: Identification based on comparison of mass spectra and CAS numbers with the NIST® 2023 library.

Table S12A. Temperature recorded under greenhouse conditions.

SEASON	Maximum temperature (°C)	Minimum temperature (°C)	Mean temperature (°C)
SPRING From March 20/21 to June 20/21	42.029 ± 0.767	8.523 ± 2.067	21.311 ± 0.423
SUMMER From June 20/21 to September 22/23	48.850 ± 1.068	9.525 ± 0.715	30.45 ± 1.898
FALL From September 22/23 to December 21/22	**50.900 ± 1.330**	7.257 ± 1.993	33.9315 ± 1.103
WINTER From December 21/22 to March 19/20	45.384 ± 1.4305	**-1.728 ± 0.290**	34.2736 ± 1.406
MEAN ANNUAL TEMPERATURE			29.991 ± 3.019

Table S12B. Humidity recorded under greenhouse conditions.

SEASON	Maximum humidity (%)	Minimum humidity (%)	Mean humidity (%)
SPRING From March 20/21 to June 20/21	69.200 ± 3.690	17.905 ± 4.342	30.347 ± 1.575
SUMMER From June 20/21 to September 22/23	**99.000 ± 0.000**	**13.000 ± 0.000**	45.500 ± 5.330
FALL From September 22/23 to December 21/22	99.000 ± 2.763	14.110 ± 1.896	25.840 ± 2.295
WINTER From December 21/22 to March 19/20	77.042 ± 2.751	16.836 ± 2.751	17.457 ± 2.030
MEAN ANNUAL RELATIVE HIMIDITY			29.786 ± 5.879

Table S13. Experimental site conditions

Parameter	Greenhouse conditions	Wild conditions
**I. Soild nutrients**		
Nitrogen (%)	0.35085 ± 0.0363	0.115 ± 0.0071
Phosphorus (mg/kg)	378.5275 ± 38.1113	75.602 ± 4.7482
Exchangeable Potassium (mg/kg)	2397.415 ± 231.9559	373.037 ± 21.5616
Exchangeable Calcium (mg/kg)	127.2802 ± 63.6401	**1506.86 ± 56.3182**
Exchangeable Magnesium (mg/kg)	318.76 ± 17.3880	**403.45 ± 6.2432**
Exchangeable Sodium (mg/kg)	133.9625 ± 29.5930	48.395 ± 5.6287
Cation exchangeable capacity (Cmol$^+$ kg)	26.485 ± 1.1355	12.005 ± 0.3448
Calcium saturation (%)	64.9775 ± 1.7150	62.5925 ± 0.6120

Magnesium saturation (%)	9.9025 ± 0.2757	**27.9875 ± 0.8669**
Potassium saturation (%)	22.9725 ± 1.2916	7.9275 ± 0.2373
Salinity by Na (%)	2.1475 ± 0.3945	1.715 ± 0.1706
Base saturation (%)	97.8525 ± 0.3945	98.2575 ± 0.1593
**II. Characterization and properties of water**		
Sand	81.04 ± 1.7321	52.32 ± 3.5360
Clay	7.6 ± 0.6855	**18.46 ±1.000**
Silt	11.3600 ± 1.3076	**29.65 ± 1.4045**
Field capacity (%)	9.3400 ± 0.6008	**19.77 ± 1.0894**
Wilting point (%)	5.0750 ± 0.3246	**10.745 ± 0.5916**
**III. Physicochemical properties**		
Moisture (%)	31.0300 ± 2.9137	2.3525 ± 0.995
Bulk density (g/cm^3)	2.1674 ± 0.0820	2.6667 ± 2.6667
True density (g/cm^3)	2.0950 ± 0.1540	2.0504 ± 2.0504
Munsell color (dry soild)	5YR 4/3	5YR 5/3
Munsell color (moist soil)	5YR 3/2	5YR 2.5/2
**IV. Habitability conditions**		
Ph	8.3365 ± 0.4867	6.9195 ± 0.0400
Electrical conductivity (dSm^{-1})	0.4110 ± 0.0364	0.2252 ± 0.0304
Oxidizable organic matter (%)	1.4554 ± 0.7277	**2.4 ± 0.03545**
Organic carbon (%)	4.0700 ± 0.4214	1.39 ± 0.2080
Ca+Mg/K ratio	3.1550 ± 0.2471	**11.43 ± 0.7783**
Ca/K ratio	2.8675 ± 0.2394	**7.91 ± 0.3281**
Mg/K ratio	0.4350 ± 0.0520	**2.2675 ± 0.2068**
Caking risk (Ca/Na)	34.1000 ± 7.0208	3.515 ± 0.4571

*The parameters evaluated were determined according to NOM-021-RENAC-2000 Specifications for sampling studies and analysis of fertility, salinity and soil classification.

Table S14. Analytical validation parameters and acceptance criteria for the HPLC-DAD quantification method of affinin.

**Parameters**	**Results**	**Criteria of acceptance**[a]
System accuracy	CV= 1.2 %	CV ≤ 1.5 %
System suitability	CV= 0.8 %, K= 4.5, R=3.2; T= 0.9; N= 6529.	CV ≤ 2%, K' > 2; *R* > 2; *T* < 2; *N* > 2000 (each injection)
System linearty	r^2= 0.99<br>IC(β_1)= 128,382-14,1873	r^2 ≥0.98<br>IC(β_1) must not include zero
Accuracy and repeatability of the method	CV= 1.3 %<br>IC(μ)= 99.7-103.6 %<br>Recovery average = 101.6 %	CV of the recovery percentage < 2%<br>IC(μ) must include 100% or that the arithmetic average of the recovery percentage is included in the interval: 98–102%
Method Linearity	r^2 = 0.99<br>IC(β_1) = 0.7-1.2	r^2 = 0.98<br>IC(β_1) must include the unit

	IC(β_0) = 5.7 – 10.9<br>$CV_{y/x}$ =1.4 %	IC(β_0) must include zero<br>$CV_{y/x}$ of the recovery percentage <2%
Intermediate precisión of the method	CV = 1.2 %	CV≤ 2%
Detection and quantification limits	LD = 1.7 µg/mL<br>LC = 5.3 µg/mL<br>r^2=0.99<br>IC(β_1) = 17,7366–91,883	r^2≥ 0.98<br>IC(β_1) must not include zero

[a]CNQFB, 2012
